# Neural Stimulation and Molecular Mechanisms of Plasticity and Regeneration: A Review

**DOI:** 10.3389/fncel.2020.00271

**Published:** 2020-10-14

**Authors:** Matthew K. Hogan, Gillian F. Hamilton, Philip J. Horner

**Affiliations:** Department of Neurosurgery, Center for Neuroregeneration, Houston Methodist Research Institute, Houston Methodist Hospital, Houston, TX, United States

**Keywords:** neurostimulation, neuroplasticity, neuromodulation, plasticity, activity-dependent plasticity, regeneration, neurotrauma

## Abstract

Neural stimulation modulates the depolarization of neurons, thereby triggering activity-associated mechanisms of neuronal plasticity. Activity-associated mechanisms in turn play a major role in post-mitotic structure and function of adult neurons. Our understanding of the interactions between neuronal behavior, patterns of neural activity, and the surrounding environment is evolving at a rapid pace. Brain derived neurotrophic factor is a critical mediator of activity-associated plasticity, while multiple immediate early genes mediate plasticity of neurons following bouts of neural activity. New research has uncovered genetic mechanisms that govern the expression of DNA following changes in neural activity patterns, including RNAPII pause-release and activity-associated double stranded breaks. Discovery of novel mechanisms governing activity-associated plasticity of neurons hints at a layered and complex molecular control of neuronal response to depolarization. Importantly, patterns of depolarization in neurons are shown to be important mediators of genetic expression patterns and molecular responses. More research is needed to fully uncover the molecular response of different types of neurons-to-activity patterns; however, known responses might be leveraged to facilitate recovery after neural damage. Physical rehabilitation through passive or active exercise modulates neurotrophic factor expression in the brain and spinal cord and can initiate cortical plasticity commensurate with functional recovery. Rehabilitation likely relies on activity-associated mechanisms; however, it may be limited in its application. Electrical and magnetic stimulation direct specific activity patterns not accessible through passive or active exercise and work synergistically to improve standing, walking, and forelimb use after injury. Here, we review emerging concepts in the molecular mechanisms of activity-derived plasticity in order to highlight opportunities that could add value to therapeutic protocols for promoting recovery of function after trauma, disease, or age-related functional decline.

## Introduction

Activity-associated plasticity refers to a form of functional and structural neuroplasticity that is driven by the depolarizing behavior of neurons, and it has been a focal area of research for the past several decades. Much of our current understanding about activity-associated plasticity has been demonstrated or discovered in experiments targeting memory, learning, and/or development (Spitzer, 2006; Feldman, 2012; Minatohara et al., 2016) Within such contexts, researchers have uncovered fundamental mechanisms governing activity-associated plasticity, including long-term depression (LTD) (Ahn et al., 1999), long-term potentiation (LTP) (Kandel, 2001), and activity-associated development of corticospinal circuitry (Martin, 2005). Still, our understanding continues to grow. Recent research has revealed a vast array of immediate early genes (IEGs), epigenetic modifiers, and even new mechanisms for transcriptional and translational regulation associated with neuronal activity (Guzowski et al., 2005; Karpova, 2014; Chen et al., 2015). Transcriptional pause-release phenomena, enhancer ribonucleic acid (RNA), and neural activity induced double-strand DNA breaks (DSBs) are just a few examples of transcriptional mechanisms that may be regulated by neural activity. Of note, DSBs can form in response to neural activity in the promoters of a subset of IEGs and can serve to enhance transcription by removing topological constraints through DNA repair of the breaks. The goal of this review is to consider these mechanisms in the context of various neural activity types, such as neuronal subtypes and their requisite receptors, the type of network in which a neuron is connected, and the pattern or duration of activity. These variables fundamentally define the differential activity-associated responses in a neuron. We recognize that in addition to those we present here, many other existing factors influence neuronal responses to changing levels of activity. For example, it is clear that metabolic and glial responses to neural activity are crucial factors (Fields and Nelson, 1992; Fields, 2008; Allaman et al., 2011; Schafer et al., 2012; Sakry et al., 2014; Jolivet et al., 2015; Kondiles and Horner, 2018); however, they are ultimately beyond the scope of this review. In addition, neuronal activity has been shown to drive neurogenesis, progenitor migration, and integration during development and in the adult hippocampus and olfactory bulb. For the purpose of this review, we focus on synaptic and network levels of plasticity rather than activity induced neurogenesis and myelination, as this topic has been well-discussed (Cao et al., 2004; Ge et al., 2007; Ma et al., 2009; Young et al., 2011; Fields, 2015; Kondiles and Horner, 2018).

**Figure 1:**
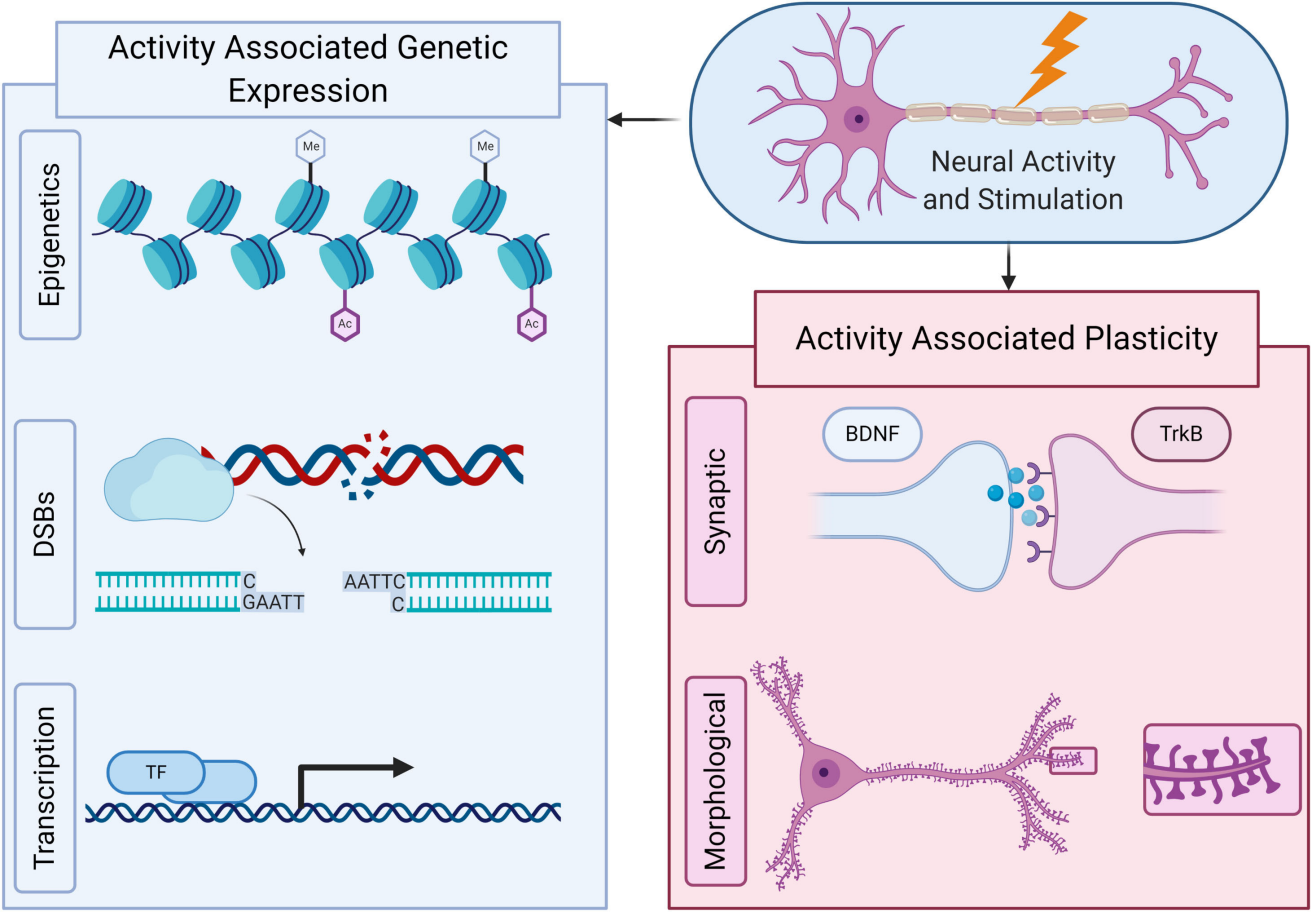
Neural activity can have broad effects on the presentation of neurons, both through altered genetic expression and changes to its structure and function. Genetically, neural activity and stimulation can cause epigenetic changes that alter the accessibility of specific genes to genetic transcription. Also, the can induce double stranded DNA breaks (DSBs) at specific sites of the genome, which can result in more stable and enhanced transcription and ultimately, through a wide variety of mechanisms including transcription factors and IEGs, can rapidly induce transcriptional changes that alter a neuron's overall genetic expression. Neural activity and stimulation can ultimately cause plasticity changes, both morphological and synaptic, through such transcriptional changes as well as in response to neurotransmitters and other signaling mechanisms.

Given the increasingly mechanistic understanding of activity-associated plasticity, it becomes clear that a review of emerging mechanisms is timely and has the potential to define new opportunities for research, collaboration, and pathways for application. However, the reader is urged to consider developmental stage, neuronal subtype, environment, and mechanism of activity in applying the concepts considered here. For example, neural subtypes express disparate receptor populations and will thus differentially integrate environmental signals. Therefore, how neurons absorb information at the cellular and circuit level is of critical importance when attempting to leverage activity-associated plasticity mechanisms in the design of experiments or applications since such experiments and applications may not be universal in outcome and application. Modification of neural activity, whether it be through chemical, electrical, magnetic, or some other means, may have unintended consequences through non-biological alteration of the microenvironment or secondary effects on the cellular or tissue target itself. Less considered aspects of activity-associated plasticity such as the duration and pattern of neuronal depolarization may play a critical role in the transcriptional profile of a neuron. Context is also critically important to consider. The environment, developmental stage, and/or presence of an injury likely alters the mechanism of activity-associated plasticity and certainly impacts the cellular response. The tools used to manipulate neural plasticity are almost as unique as the neuronal circuits they target and must be carefully considered alongside the environmental milieu. Hence, this review will cover relevant mechanisms of activity-associated plasticity and potential considerations for harnessing them to promote regeneration and restoration of function. Neural activity can mediate plastic responses through several intrinsic and extrinsic vectors. Additionally, by manipulating activity patterns, it may be possible to repair a degenerated or damaged brain or spinal cord (Figure 1). We will: (1) consider the differences between exogenous and endogenous modulation of neural activity; (2) provide a brief overview of mechanisms of neuronal activity-associated plasticity, such as brain derived neurotrophic factor (BDNF) and its receptors, trophic modifiers, IEGs, epigenetics, and genetic regulation; (3) present research demonstrating robust transcriptional alterations to changes in intrinsic neuronal activity patterns; (4) review potential therapeutic methods to leverage these mechanisms to promote recovery; and (5) explore how different types of rehabilitation and stimulation differentially affect these mechanisms and potentially recovery.

## Exogenous vs. Endogenous Modulation of Neural Activity

The distinction between changes in neural activity initiated by exogenous stimulation through an electrode, magnetic coil, or some such device compared with those brought on by behavioral activity or endogenous stimuli is a crucial one (Linderoth and Foreman, 1999; Stagg and Nitsche, 2011; Young et al., 2011; Kravitz and Bonci, 2013). Endogenous stimulation induced by physical activity or sensory stimulation initiates neural activity through physiological changes in local ion gradients caused by synaptic activity or environmental sensing receptors. Exogenous stimulation causes local changes in ion gradients in a non-physiological and larger scale mode when applied magnetically or electrically. While this type of stimulation arguably initiates normal depolarization of a neuron, it may also affect other systems and cause non-physiologic responses. Although the end result can be similar, the question remains as to whether exogenous stimulation is truly synonymous with endogenous or natural activation of neurons given that exogenous stimulation methods may indirectly manipulate local ion gradients and initiate other changes not demonstrated through natural activation of neural pathways. Still, research has shown that exogenous stimulation can induce spike-timing-dependent plasticity through closed loop stimulation coincident with voluntary behavior or endogenous stimulation in rats (Rebesco et al., 2010) and non-human primates (Zanos et al., 2011). Thus, tools to manipulate neural activity can impact the plastic rewiring of neural circuits. Importantly, altered activity patterns can induce strengthening or weakening of connections between two neural circuits independent of voluntary action, as demonstrated by activity-dependent strengthening or depression between sites in the sensorimotor cortex of awake behaving primates via paired stimulation (Seeman et al., 2017). A host of tools have been developed to manipulate neural activity, and many have relevance in a clinical context. Notably, neural activity modulating therapies have emerged with varying degrees of success (Mailis-Gagnon et al., 2004; Williams et al., 2010; Harkema et al., 2011; Sayenko et al., 2019).

Furthermore, there is a considerable nuance in activity-associated plasticity, particularly in the context of extrinsic factors, such as injury and the environment. While much is known about activity-associated modifiers in specific frameworks (e.g., learning, memory, and principles of Hebbian plasticity), exciting research has surfaced that illustrates the importance of neuronal cell type (Dehorter et al., 2015) and temporal firing patterns (Fields et al., 2005; Lee et al., 2017; Tyssowski et al., 2018) on activity-associated responses relevant to regeneration. After spinal cord injury (SCI), the spinal cord environment is largely changed; thus, results from healthy spinal cord experiments may not be predictive of activity-evoked plasticity effects in models that exhibit gliosis, neuronal atrophy, inflammation and altered channel distribution or function. For example, following injury, gamma-aminobutyric acid (GABA)-ergic neurons reduce their expression of the membrane-bound potassium-chloride co-transporter 2 (KCC2), which shuttles chloride outside of the cell. This imbalance, which is not present in the healthy spinal cord, causes a shift in membrane polarization, resulting in normally inhibitory GABAergic cells becoming excitatory. Interestingly, this shift after injury is a recapitulation of development where NKCC1 cotransporters appear earlier than KCC2 cotransporters, allowing preferential flow of chloride into GABAergic neurons, and in this developmental state GABAergic neurons exhibit excitatory behavior (Medina et al., 2014). KCC2 reduction has been shown to occur within 24 h of injury and is associated with maladaptive plasticity in chronic SCI (Grau and Huang, 2018). This suggests that injury can alter the excitatory/inhibitory balance through this conversion and disrupt normal circuit function (Huang et al., 2016). In addition, injury to the central nervous system (CNS) can differentially affect how BDNF regulates KCC2 expression in spinally transected and intact rats. For instance, BDNF normally downregulates KCC2, producing an increase in central sensitization. In contrast, following transection injury, BDNF causes an increase in membrane bound KCC2, thereby restoring the inhibitory action of GABAergic neurons, leveling the excitatory/inhibitory balance and decreasing central sensitization (Huang et al., 2017). The variability in pathological states of neural circuitry as well as the unique mechanisms of unique modes of neuromodulation are important considerations for not only the interpretation of current literature, but also a challenge to the design of future, mechanistic studies.

## Mechanisms of Neuronal Activity-Associated Plasticity

Activity-associated plasticity has long been linked with development; however, such mechanisms may also be relevant in the adult nervous system. Post-mitotic neurons in the brain and spinal cord, long thought to be generally stable in structure and nature, have since been proven to adapt significantly in response to neuronal activity, exhibiting changes in morphological properties, gene expression, and synaptic strength. Further, neuronal firing behavior has been shown to influence a host of mechanisms from synaptic plasticity (Davis, 2006) to dendritic arborization (Kellner et al., 2014) and even to neuronal regeneration (Elzinga et al., 2015; Chan et al., 2016). Such mechanisms are naturally governed by the intrinsic signaling of molecular factors that have been, and continue to be, increasingly illuminated over the past several decades. A host of intrinsic and extrinsic factors exhibit direct activity-associated expression changes and have broad effects on neuronal expression, such as BDNF, neuronal PAS domain protein 4 (Npas4), and many others. Recent literature describes neuronal activity-associated gene expression control mechanisms, including a potential role for activity induced DSBs and RNA pause-release, alluding to the depth and breadth of complexity of activity-associated gene expression (Madabhushi and Kim, 2018). A full understanding of the ways in which neuronal activity influences gene expression may prove critical to developing potential neuronal activity modulation therapies. We here highlight some well-documented and some newly-discovered fundamental mechanisms for intrinsic neural control of molecular behavior and signaling in response to neural activity.

### BDNF and Neuronal Plasticity

BDNF and its receptor2, TrkB, have been widely studied in both the CNS and the peripheral nervous system (PNS) (Nagappan and Lu, 2005; Nagahara and Tuszynski, 2011). BDNF is a powerful neurotrophic factor known to induce plastic changes that regulate neuronal growth, excitability, and even regeneration (Jin et al., 2002; Garraway and Huie, 2016). A major mechanism of action for BDNF is via the binding of TrkB. This binding initiates a signaling cascade that regulates activity-associated dendritic plasticity in mice (Lai et al., 2012) as well as local production of proteins associated with synapse formation (Lu et al., 2008). Dendritic TrkB is upregulated in an activity-associated manner and inserted specifically at targeted dendrites, explaining how a diffusible molecule (e.g., BDNF) can initiate local changes in synaptic plasticity (Tongiorgi et al., 1997; Nagappan and Lu, 2005). Importantly, field electric stimulation has been shown to influence the trafficking of TrkB from the intracellular pool to the membrane of neuronal processes in cultured hippocampal neurons (Du et al., 2000).

BDNF is one of a few neurotrophic factors that exhibit activity-associated behavior, with activity-associated mechanisms governing BDNF transcription, dendritic targeting, and trafficking of BDNF protein, and messenger RNA (mRNA) release and conversion of BDNF to a mature form (Jin et al., 2002; Zhou et al., 2006; Im et al., 2010; Bading, 2013; Palomer et al., 2016). BDNF is widely implicated as a major driver of neuronal plasticity that directly contributes to learning. For example, TrkB signaling is required for spatial memory formation in mice (Lai et al., 2012), BDNF signaling contributes to the conversion of early to late phase long-term memory (Lu et al., 2008), BDNF signaling facilitates cocaine-seeking addictive behavior in mice (Im et al., 2010), and BDNF administration has even been implicated in functional recovery after stroke (Berretta et al., 2014) and SCI (Ghosh et al., 2018) in rodents.

Critical reasons for considering BDNF and its effects under the umbrella of neural stimulation are: (1) BDNF is a potent regulator of neural plasticity, including downstream enhancement/modulation of synaptic plasticity (Karpova, 2014), cell survival, and morphological properties of neurons (Zagrebelsky and Korte, 2014) and (2) BDNF activity is associated with levels of neural activity. Synaptic plasticity is largely regulated by, and sometimes dependent on, BDNF signaling (Lu et al., 2008). In fact, BDNF is thought to be synthesized in dense core vesicles and secreted at the synapse in response to neuronal activity. Importantly, new evidence tracking BDNF tagged with quantum dots in hippocampal cultures suggests a mechanism for activity-associated release of BDNF from endocytosed vesicles in the post-synaptic dendrite (Wong et al., 2015), indicating multiple sources and mechanisms exist to regulate activity-associated release of BDNF at synapses. *In vitro* experiments using mouse hippocampal cultures at different developmental time-points highlight the importance of neuronal activity on BDNF's structural effects on neurons. In loss-of-function experiments, blocking endogenous BDNF resulted in hippocampal neurons with less dendritic complexity and longer dendritic spines with thinner heads (Kellner et al., 2014). Exogenous application of BDNF in culture did not significantly alter dendritic structure or density as reported in *in vivo* experiments (Ji et al., 2010); however, cultures with reduced levels of spontaneous activity were responsive to exogenous BDNF and exhibited increased dendritic density, indicating that levels of neural activity may play an important role in the neuronal response to BDNF (Kellner et al., 2014). Overall, these studies show that BDNF regulation is quite complex, and that the effects of BDNF include modulation of synaptic and dendritic structure as well as network plasticity; but, they are ultimately context and activity specific.

Observations on the effects, control, and pharmacological application of BDNF in disease not only have led to an appreciation of its importance, but have also raised questions about the mechanisms whereby levels of BDNF are modulated in the spinal cord. In patients with incomplete SCI, a graded-intensity, locomotor exercise regimen increased serum levels of BDNF. Still, said levels were related to the intensity of the locomotor activity rather than the activity itself (Leech and Hornby, 2017). It remains unclear in injury models to what extent such recovery is dependent on neural activity-associated plasticity vs. metabolic mechanisms. Rodent work indicates that voluntary wheel running increased the expression of several downstream effectors for the action of BDNF on synaptic plasticity [e.g., synapsin I and growth-associated protein (GAP-43) mRNA] (Gómez-Pinilla et al., 2002). Further, voluntary wheel running increased production of the ketone body β-hydroxybutyrate in the liver, which is both an energy source used in the brain and an inhibitor of class 1 histone deacetylases (HDACs) (Sleiman et al., 2016). HDACs cause the deacetylation of histones, resulting in less accessible and more tightly bound DNA. HDAC inhibition increases levels of synaptic plasticity genes, including cAMP response element-binding protein (CREB), BDNF, and calmodulin-dependent kinase II (CaMKII) (Guan et al., 2009; Koppel and Timmusk, 2013). Thus, production of this ketone could explain some of the beneficial effects of exercise. In fact, application of β-hydroxybutyrate to cultured cortical neurons, hippocampal slices, and via *in vivo* intraventricular injection all resulted in elevated expression of BDNF transcripts (Sleiman et al., 2016). Therefore, exercise-associated BDNF production may not directly rely on intrinsic firing of neurons. Rather, it may be a passive result of metabolite production.

BDNF appears to be a potent regulator of neural plasticity; however, some studies have tempered enthusiasm for a potential BDNF panacea-type therapy to injury and disease due to its possible pronociceptive effects, particularly following CNS trauma (Garraway and Huie, 2016). For instance, Grau and Huang (2018) have demonstrated that BDNF is necessary and sufficient to enable spinal learning of a shock withdrawal response in a thoracic transection model in rats. Yet, BDNF also appears to play a critical role in the formation of neuropathic pain after injury (Smith, 2014; Garraway and Huie, 2016). Given the broadly acting nature of BDNF (Karpova, 2014) and that its effects are context dependent (Xiao et al., 2009), it seems logical to conclude that other activity-associated mechanisms may play a role in regulating neural behavior and in modulating the effects of BDNF itself.

### Trophic Modifiers of Neuronal Complexity and Excitability

Aside from BDNF, other trophic factors are modulated or released in an activity-dependent manner. For instance, neuritin mRNA was initially identified as having activity-associated transcription and purified neuritin and was later revealed as a modulator of neurite outgrowth and arborization in embryonic hippocampal and cortical mouse neurons (Naeve et al., 1997). Subsequent studies have demonstrated that neuritin induces neuritogenesis and that it plays a role in the maturation and stability of synapses while also increasing neurotransmitter release in cortical neurons (Yao et al., 2018). Neuritin activity can also be mediated through nuclear factor of activated T-cells cytoplasmic 4 (NFATc4) and calcineurin (CaN). Neuritin induces neurite morphological changes through calcium signaling by upregulating L-type voltage gated calcium channels (Zhao et al., 2018). Additionally, the trophic factor fibroblast growth factor (FGF) appears to mediate the activity-dependent neurite morphological effects of neuritin. Inhibition of the FGF receptor attenuates the effects of neuritin on neurite arborization and complexity, indicating that other trophic factors play a role in activity-associated effects, albeit indirectly (Shimada et al., 2016). Neuritin and FGF are not the only identified trophic factors associated with neuronal activity. Isoforms of the *homer1* gene were found to express activity dependent expression and Homer1a, a product of the *homer1a* IEG, was found to modulate pre- and post- synaptic remodeling in glutamatergic neurons in a biphasic activity induced manner (Xiao et al., 2000; Inoue et al., 2007). Further, other products of the *homer1* gene, Homer 1b/c, are spinal synaptic scaffolding proteins in the post-synaptic density of excitatory synapses. Indeed, work from Yao et al. demonstrates that Homer 1b/c not only regulates CREB phosphorylation and c-fos activation in the spinal dorsal horn, but that it may play a role in the formation of chronic pain following spinal injury (Yao et al., 2014).

### Neural Activity and IEGs

IEGs are genes that exhibit a rapid and transient change in expression in response to a variety of extracellular stimuli in a protein synthesis independent manner. In the context of neural activity, IEGs are a proposed mechanism for rapid, functional translation of altered depolarization behavior (Flavell and Greenberg, 2008). A host of genes have been identified as IEGs and are upregulated/downregulated in response to changes in neural activity at early time points after stimulus administration (Carulli et al., 2011). In some cases, IEG responses mediate transcriptional factors, including growth arrest and DNA damage inducible β (Gadd45β), Npas4, early growth response 4 (Egr4), nuclear receptor subfamily 4 group a member 1 (Nr4a1), Fos, and many others (Spiegel et al., 2014). IEGs exhibit transcriptional changes immediately following an altered period of activity and the full time-course of this response can range from hours to days (Morgan and Curran, 1989). For instance, increased transcription of c-fos occurs within 5 min of induction (Sheng and Greenberg, 1990). Recently, it was demonstrated that some IEGs exhibit control mechanisms that are not directly dependent on neural activity (Okuno, 2011; Bahrami and Drabløs, 2016). While Arc expression is normally tightly associated with neural activity initiated through behavior or sensation, it has been shown that Arc is decoupled from activity patterns in novel recognition paradigms. For example, control mice placed in a novel environment exhibit increased Arc expression in their hippocampi. However, Arc levels are reduced following lesions to the fornix (Fletcher et al., 2006) and following repeated environmental exposure (Guzowski et al., 2006). Fornix lesions did not impact overall activity levels in the hippocampus and overall hippocampal electrophysiology remained unchanged throughout the study. This indicates that there is a secondary regulator of Arc expression in the hippocampus beyond neural activity alone. Both the target responsiveness to an IEG and the environment of the circuit and organism are critical considerations for manipulating IEG expression to promote activity-dependent plasticity in a clinical context.

IEGs have been identified as a potential primary mechanism for modulating and maintaining neural connectivity, and they may have mechanisms for neuron-to-neuron signaling in an activity-associated manner. Arc protein has recently been shown to form a structure reminiscent of a Gag capsid. This capsid-like structure is secreted from neural cells and can inject mRNA into recipient cells, which can then exhibit activity-associated translation (Pastuzyn et al., 2018). This striking example of neuron-to-neuron signaling highlights the importance of considering activity-associated signaling at the circuit level since modulation of activity in a single neuron can initiate changes in transcription and translation in other neurons. The full extent to which this extrinsic signaling is relevant remains unknown. Therefore, future research must explore this further to better understand how neuron-to-neuron signaling mediates plasticity following bouts of depolarizing activity.

### Activity-Associated Plasticity in Epigenetics and Gene Expression

How environmental stimuli cause lasting adaptive changes (i.e., the formation of memory) has been an area of study since Flexner et al. first established that new protein synthesis was necessary for the formation of adaptive behavior, namely memory, in a short window following an environmental stimulus (Flexner et al., 1963). Specifically, bilateral injections of puromycin, a protein synthesis inhibitor, into the hippocampi and adjacent temporal cortices of mice abrogated conversion of memory from short- to long-term in a simple maze learning paradigm, indicating that a protein was necessary for the formation of long-term memory in mice. The model of transcription and translation at the time did not include a mechanistic explanation for the formation of adaptive behavior in response to environmental stimuli. This led researchers to investigate how exactly adaptive behaviors are formed. The eventual discovery of transcription factors and promoter regulatory elements explained how gene expression might vary following exposure to external stimuli. Particularly, in neural activity coupled gene expression, serum factors, and upstream non-coding promoter regulatory elements were both found to produce rapid changes in transcription following bouts of neural activity. For instance, calcium induction through voltage gated channels was found to be the driving event for induction of *c-fos* expression in neurons (Morgan and Curran, 1986). Such expression required a particular sequence upstream of the promoter (Sheng et al., 1988), which was bound by CREB. CREB was eventually discovered to be a transcription factor that stabilized expression of *c-fos* when phosphorylated by calmodulin dependent kinase (Sheng et al., 1991). Together, these discoveries describe a mechanism whereby external stimuli such as neural depolarization can cause changes that initiate a signaling cascade capable of altering transcription factors, which may then affect a transcriptional response by binding to transcriptional regulatory elements and stabilizing or inhibiting transcription. Still, such observations do not describe how lasting gene expression changes could occur in response to environmental stimuli. With demonstrations that histone methylation causes conformational chromatin changes, which decrease gene expression at the site of methylation (Rea et al., 2000) and that acetylation results in looser chromatin structures with enhanced transcription at the site of acetylation (Brownell et al., 1996), researchers discovered mechanisms whereby environmental influences could affect stable changes to gene expression. Epigenetic modifiers were linked to neural activity (Qiu and Ghosh, 2008) and have since been considered critical in describing activity-associated changes in neural cells (Carulli et al., 2011; Ciccarelli and Giustetto, 2014; Karpova, 2014). Through epigenetic modifications, environmental triggering of signaling cascades, and transcription factor binding to promoter regulatory elements, it is clear that neural activity patterns can cause both temporary and stable changes to neural transcription.

In addition to transcription factors binding to regulatory elements, novel mechanisms for transcriptional regulation have been uncovered. Gariglio et al. found an unanticipated clustering of RNA polymerase II (RNAPII) at the 5′ end of the β-globin genes in mature erythrocytes in transcriptional run-off assays, which they posited may constitute a rate-limiting step to transcription (Gariglio et al., 1981). Initially identified in metazoan model systems, researchers have uncovered a mechanism of RNAPII pause-release that is widespread and that not only regulates rates of transcription, but also is subject itself to regulation (Adelman and Lis, 2012; Jonkers and Lis, 2015). Chromatin immunoprecipitation sequencing (ChIP-seq) revealed RNAPII binding at promoters of various IEGs under basal conditions (Kim et al., 2010). Researchers have directly identified transcriptional pause-release regulation of c-fos and Arc, and it is likely that activity-associated signaling pathways regulate this pause-release phenomena (Kim et al., 2010; Schaukowitch et al., 2014; Joo et al., 2016). Continually, it has been posited that enhancer RNA may be recruited to IEG promoters and initiate RNAPII unpausing in an activity-associated manner (Madabhushi and Kim, 2018). Recent studies have revealed that pausing of RNAPII positively correlated with tri-methylation of the 27th lysine residue at histone 3 (H3K27me3) in developing mouse cortical neurons (Liu et al., 2017), indicating a role of pausing in fate selection and expression during development. Enhancer RNA (eRNA) have been shown to trigger transition of RNAPII from a paused to active state by facilitating release of the negative elongation factor (NELF) complex from target promoters following IEG activation (Schaukowitch et al., 2014). RNA pause-release may thus be a key transcriptional regulator of neural activity-associated gene expression and may describe yet another method whereby neural depolarization causes changes in gene expression.

Beyond pause-release, the discovery of neural activity-induced DSBs indicates a startling potential mechanism for regulating transcription (Madabhushi et al., 2015). DSBs are generally thought to be a destabilizing and cytotoxic event. Neural activity has been found to cause DSBs both *in vitro* (Crowe et al., 2006) and *in vivo* (Suberbielle et al., 2013); and, neural activity-induced DSBs may be used as a method to regulate activity-associated transcription (Madabhushi and Kim, 2018). Researchers have demonstrated that neural activity-induced DSBs form within the promoters of several IEGs (e.g., Fos, Npas4, and Egr1) and that said DSBs are sufficient to enhance transcription (Bunch et al., 2015; Madabhushi et al., 2015). Neural activity-induced DSBs are generated by topoisomerase IIβ and are stabilized rather than rapidly repaired. Normally, topoisomerases cause transient breaks in DNA to relieve strain caused by DNA processing activity. They rapidly repair DSBs, allowing the breaks to avoid detection by DNA damage response pathways. In contrast, neural activity-induced DSBs are longer lasting and are somehow stabilized, though the mechanisms remain unclear. Activity-induced stable DSBs are then detected by DNA damage repair pathways, which can result in enhanced transcription (Madabhushi et al., 2015). Recently, a member of the growth and arrest DNA damage family, growth arrest and DNA damage inducible gamma (GaDD45y), was identified as mediating fear memory consolidation in mice through binding at DSBs in the promoter regions of several plasticity related IEGs (Li et al., 2019). More research is needed to fully understand how DSBs form and whether different types of activity may influence their activity. Still, these startling observations hint at yet another mechanism in the complex and layered activity-associated regulation of neural plasticity.

### Temporal Patterns of Activity and Gene Expression

The wealth of transcriptional and translational regulatory mechanisms sensitive to neural activity describe a layered and complex system. Clearly, activity-associated plasticity occurs. Newly discovered mechanisms indicate that the pattern and length of neural activity trains produce distinct changes in transcription. Further, they hint at a potentially subtle and temporally regulated interaction between activity and transcriptional and translational response. Indeed, patterns of neural depolarization and the duration of a depolarization train may initiate unique transcriptional responses. Enticing new research in mouse DRG neurons examined genes regulated by activity patterns and timing (Lee et al., 2017). While previously identified activity-associated master regulators such as BDNF, EGR4, Gadd45b, Npas4, and Nr4a1 were generally found to be differentially controlled by stimuli, independent of pattern or time, a host of novel RNA transcripts whose levels depended explicitly on temporal patterns of activity in mouse DRG neurons were determined (Lee et al., 2017). Pathway analysis of the observed transcripts revealed different patterns and total durations of stimulation can induce activity-associated changes in NGF and Rac signaling, important modulators of neurite outgrowth. Electrical stimulation of mouse DRG neurons using different patterns, but the same total number of electrical pulses at 10 Hz, revealed network changes in gene expression dependent on patterned stimulation (Lee et al., 2017). Excitingly, many of the novel transcripts exhibited changes in transcription levels when DRG neurons were paced with different patterns of stimulus, thus classifying them as activity-associated and suggesting their actions affect neuronal plasticity and growth. The observation that patterned activity can alter gene expression, while preliminary, has vast implications on neuronal response to stimulation. If patterns of activity have broadly differential effects on neuronal transcripts, then the implications for clinical stimulators would be profound. Driving specific patterns of activity in targeted brain and spinal circuitry may thus be a way to control transcriptional response and cause desirable expression changes to promote recovery following CNS trauma. Work by Tyssowski et al. (2018) further supports the hypothesis that there are temporal signatures of neural response to activity and that sustained vs. brief depolarizations result in large differentials in RNA transcripts. By manipulating the duration, but not frequency, of neuronal activity, Tyssowski et al. identified three functionally distinct transcriptional patterns dependent on unique regulatory pathways comprising exclusive waves of transcription that occur in response to patterned neural activity. These examples are the first to show not only the importance of duration and temporal pattern of neuronal activity on transcriptional output, but also how neural firing patterns may cause large changes in neuronal transcription. It has yet to be determined whether frequency of stimulation may play a role, or whether these effects are restricted to mouse DRG neurons and mouse and rat cortical neurons. Still, these observations may prove critical when considering how to modulate neuronal activity to enhance recovery. In particular, stimulation regimes for exciting the brain or spinal cord through optogenetic, electrical, or magnetic stimulation are often static and periodic in nature.

It is critical to examine how dosing and pattern play a role when stimulating the brain and spinal cord. Work by Taccola et al. demonstrates that locomotive central pattern generators in the rat spinal cord are optimally activated by noisy dorsal root stimulation patterns (Taccola, 2011). Such an observation may have implications for existing clinical spinal stimulators given that introduction of randomness could improve the efficacy with which central pattern generators are recruited during spinal neuromodulation. Still, this begs the question of whether overstimulation or random stimulation may induce negative effects. After spinal injury, there is a known excitatory inhibitory imbalance that drives additional activity for prolonged periods, and thus may encourage the onset of spasticity and neuropathic pain through inadvertent maladaptive plasticity (Lavrov et al., 2008; Ferguson et al., 2012; Grau et al., 2017). Therefore, it is crucial to minimize the risks of stimulation induced maladaptive plasticity through optimal dosing and careful targeting of select circuits to minimize secondary effects while maximizing the benefits. While random stimulation paradigms may more effectively enhance recruitment of motor associated dorsal roots, it remains to be determined whether such paradigms may also more effectively enhance maladaptive plasticity (Lavrov et al., 2008).

Given the observation that pattern and duration of neural activity can each greatly alter the functional transcripts of a neuron, it seems clear that stimulation paradigms must be more closely considered. It is no surprise that most reported *in vivo* CNS stimulation paradigms have been static in nature. *In vivo* injury and stimulation experiments are already costly, difficult, and complex enough without the addition of stimulation regime variables, such as pattern and duration. It seems imperative that future research includes a characterization, whenever possible, of the activity dependent transcriptional changes relevant to injured circuitry and the response of the damaged nervous system to changes in neural activity. Given the cost, time, and importance associated with neural injury and neuromodulation studies, the field would greatly benefit from shared resources such as a tissue repository or transcriptional database to enhance our ability to leverage activity associated mechanisms to initiate recovery.

## Leveraging Neuronal Activity-Associated Mechanisms to Promote Recovery

**Figure 2:**
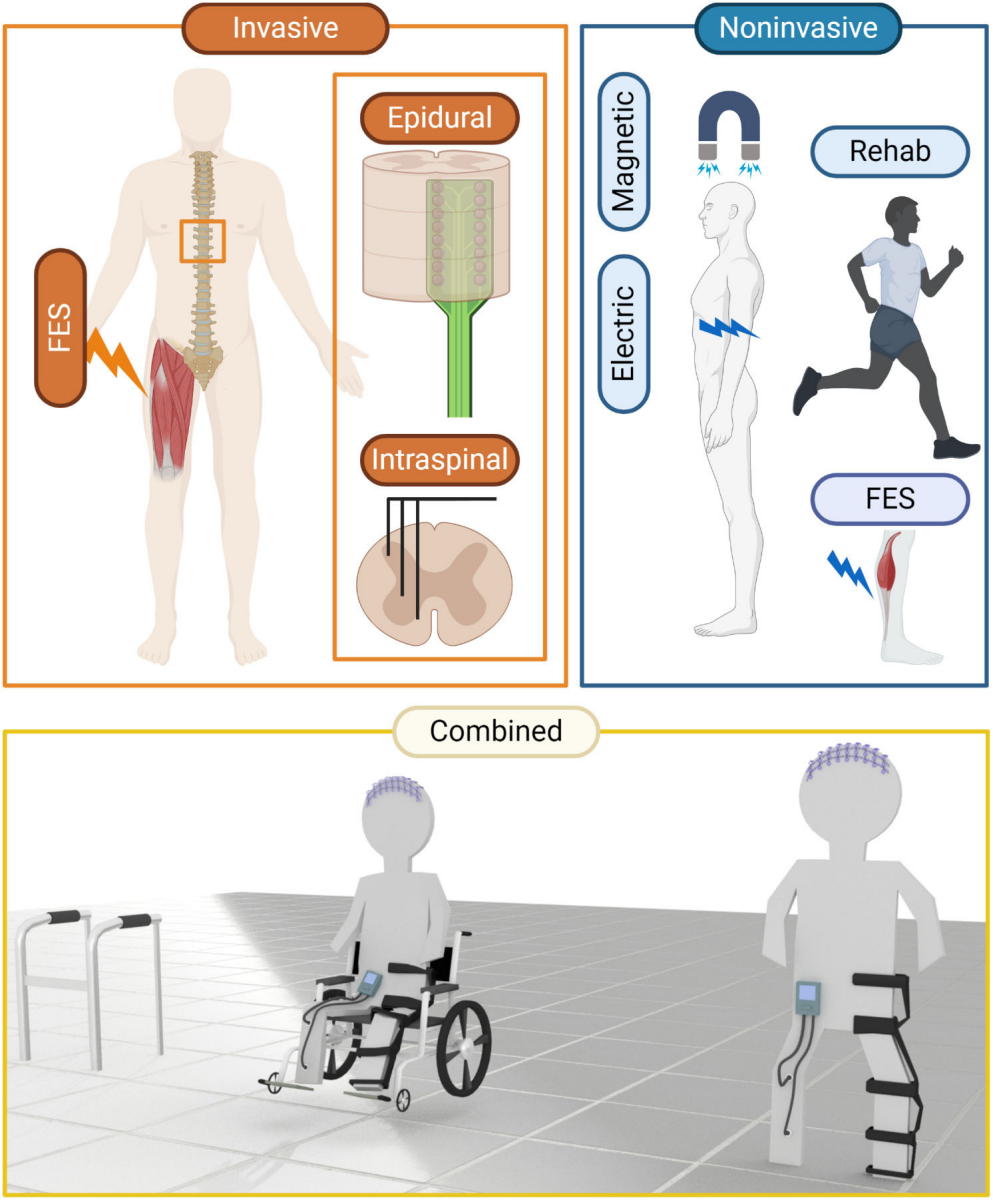
Clinical means to alter neural activity have been employed with a view to restore or improve function after insult. Invasive techniques, typically involving surgical placement of electrodes to control muscle or neural activation have been used for pain management and functional restoration after neural trauma or stroke. Non-invasive techniques, such as TES, TMS, FES, and rehabilitation are more clinically palatable, though non-invasiveness is generally correlated with a decrease in precision. An exciting area is emerging where techniques are being combined with greater effect. Invasive and non-invasive means to record and manipulate neural activity are being combined to “close the loop” and restore previously intractable function.

Neuronal activity-associated plasticity mechanisms play an important role in recovery following damage to the PNS and/or CNS through trauma, stroke, and neuroregenerative disease. Given the array of methods currently in use or being developed for manipulating neuronal activity following injury, it may be relevant to consider our understanding of activity-associated plasticity in the context of potential clinical applications. Following damage to the nervous system, some evidence suggests that regrowth and reconnection of axons occurs in the PNS, while very limited evidence indicates that such plasticity occurs in the CNS (Liu et al., 2011). The success of physical rehabilitation, electrical stimulation, and other therapeutic strategies to mitigate CNS and PNS damage from trauma, stroke, or neurodegenerative disease indicates a possible role of activity-associated plasticity in recovery following damage. Here, we present some of the clinical and potentially translatable methods to manipulate neuronal activity, and we highlight potentially relevant considerations (Figure 2).

### Passive and Active Exercise Are Effective Tools for Initiating Recovery After SCI

Two common rehabilitation tools are (1) passive exercise, which requires no controlled physical effort, and (2) active exercise, which involves subjects performing assisted or unassisted movements using volitional control. After spinal injury, a lack of supraspinal input can enhance sensory control of spinal circuitry. Stretching of a skeletal muscle activates the H reflex through group Ia afferents, which can lead to changes in sensory driven reflexes (Skinner et al., 1996; Gazula et al., 2004). Repetitive passive conditioning of limbs after spinal injury has been shown to “normalize” reflex responses in absence of supraspinal input in both rodents (Skinner et al., 1996) and humans (Rösche et al., 1997; Kiser et al., 2005). This can reduce spasticity and habituation of somatosensory reflexes. However, continued passive conditioning is necessary to maintain such effects, at least in humans. Passive bicycling in spinally transected rats led to an upregulation of neural activity-associated proteins, BDNF, and adenylate cyclase 1 (ADCY1) in the somatosensory cortex, and said upregulation was accompanied by an increased tactile response in the denervated limb (Graziano et al., 2013). This indicates that, at least in rats, passive exercise can facilitate cortical plasticity through activity-associated mechanisms, though the degree to which passive exercise alone leverages permanent cortical changes in humans remains unclear. Other studies have demonstrated that active exercise also produces neuronal plasticity, which can lead to functional recovery in both humans (Jurkiewicz et al., 2007) and rats (Kao et al., 2011). Many active exercise-associated functional plasticity studies have exhibited a task-dependent and/or task-specific plasticity that is associated with particular movements, indicating that movement-associated circuitry should be targeted in rehabilitation therapy (Lynskey et al., 2008). For example, one case report revealed that a person with cervical injury who received bimanual somatosensory stimulation in combination with massed practice (repetitive task oriented training) manifested enhanced sensation, grip force, and performance of task-specific hand skills, such as writing, page turning, lifting of a small object, manipulating checkers etc., which coincided with a reorganization of the cortical map (Hoffman and Field-Fote, 2007). Such cortical rewiring suggests active exercise can initiate a type of synaptic plasticity that is driven by activity-associated mechanisms. The improvement of task-specific movements and enhanced sensation through passive and active exercise following disruption of the CNS indicate plasticity plays a role, possibly through activity-associated mechanisms, in the restoration of motor function following injury. In passive exercise regimens, such as on a fixed bike, functional recovery is dependent on continued input. It is believed that load sensing sensory afferents cause plastic changes to occur in central pattern generators located in the spinal cord (Dietz and Harkema, 2004). Alternatively, active exercise (i.e., voluntary or assisted standing/walking, treadmill training, and forelimb reaching) can produce reorganization of cortical and spinal circuitry, resulting in improved motor function. Yet, the degree to which such plasticity is sensory load-dependent or can be mediated by other forms of propriospinal input remains unknown (Lynskey et al., 2008).

Clearly, exercise can initiate plastic changes in the CNS, but through what activity-associated mechanisms is this achieved? A myriad of reports haven examined the benefits of exercise on the brain. The hippocampus in particular has been proven to exhibit significant and long-lasting changes in response to increased levels of physical activity (Rendeiro and Rhodes, 2018). BDNF levels increase in the rat brain following bouts of elevated physical activity (Marais et al., 2009; Rasmussen et al., 2009). These increased levels are thought to occur, in part, due to muscle derived factors such as PGC-1α and its downstream protein FNDC5, both of which were found to be elevated in the hippocampus of exercising mice (Rendeiro and Rhodes, 2018). Circulating FNDC5 is believed to cross the blood brain barrier and directly modulate hippocampal gene expression. Therefore, a factor released from skeletal muscle during exercise can effect changes in the brain. Interestingly, neural activity can also effect changes in skeletal muscle by initiating transcriptional changes in the muscle through HDAC4 signaling at the neuromuscular junction (NMJ) (Cohen et al., 2007). HDAC4 is normally localized at the NMJ; however, following reduced neural input through denervation, HDAC4 is released and exhibits activity dependent transcription in the muscle. Consequently, there are bidirectional control mechanisms whereby neural activity can initiate transcriptional changes in the muscle and altered muscle activity can influence transcription in the brain. Stimulation, particularly in the spine, can initiate motor responses and thus cause changes to the neuromuscular axis. Therefore, it is critical to consider the broad physiological responses initiated by spinal stimulation or physical activity. Such changes initiated by physical activity are not limited to effects induced by the muscle alone. Exercise also causes changes in peripheral organs, which may in turn produce paracrine factors capable of initiating transcriptional changes in the CNS. Recently identified metabolic factors such as β-hydroxybutyrate may contribute to changes in BDNF expression in the hippocampus, possibly describing a mechanism where exercise influences learning, memory, and mood (Chen et al., 2011; Sleiman et al., 2016). Importantly, serum levels of BDNF in humans with spinal injury have been positively correlated with exercise intensity (Leech and Hornby, 2017). These observations argue for a potentially metabolic mechanism for BDNF release and activity, and this may be the case particularly in facilitating supraspinal plasticity. There are a host of ways in which exercise and activity influence neural plasticity in the brain and spinal cord, both in the long- and short-term. Bouts of physical activity can transiently enhance blood brain barrier permeability, serum levels of BDNF and VEGF, neuronal IGF-1 uptake, and VEGF expression in the hippocampus as well as initiate long term cardiovascular changes and neuroplastic rewiring (Stimpson et al., 2018).

The observation that BDNF and other neurotrophin levels are locally modulated by neural activity is further supported by changes in neurotrophin levels at the spinal level following different exercise regimens. For example, Côté et al. (2011) examined how passive cycling and treadmill stepping rehabilitation may contribute to activity-associated plasticity after complete thoracic spinal trans-section in rats. They found that both stepping and cycling promoted increased levels of BDNF, neurotrophin-3 (NT-3), and neurotrophin-4 (NT-4) at the lumbar enlargement of SCI rats. The increased levels were positively correlated with recovery of spinal H-reflex responses. Further, the researchers demonstrated that while both exercise regimens promoted enhanced levels of glial derived neurotrophic factor (GDNF) rostral to the injury site, only step-training promoted elevated levels of GDNF at the lumbar enlargement, indicating that sensory feedback may play a critical role in spinal neurotrophic expression. This experiment provides significant evidence that different physical movements may involve unique spinal networks. Further, spinal network activity may contribute to unique molecular changes post-injury that may be relevant in the selection of rehabilitation regimens.

One major barrier in developing effective rehabilitation exercises to promote functional improvement stems from limited control of neural circuitry following CNS injury. Numerous efferent fibers and interneuron circuits that could be manipulated to provide beneficial plastic changes may exist; however, it is also possible that said fibers and circuits are not accessible through exercise. There are means to manipulate neural circuitry purely through exercise and movement-based therapy (e.g., stretch receptor activation and supraspinal/cortical activation through voluntary intent and propriospinal activation); however, the types and patterns of activation are limited to physiological responses under fixed conditions. A further barrier is the degree to which voluntary control is possible after damage. In active exercise, the ability to exert supraspinal control is important and limitations in volitional control minimize the abundance and types of neurons that can be activated. Still, physical rehabilitation remains the most effective and only accepted therapy following damage to the CNS and PNS.

### Electrical Stimulation of the CNS and PNS

Given the limits of rehabilitation, researchers have explored techniques to actively manipulate neural activity within the spinal cord. Direct application of an electric current via penetrating or non-penetrating electrodes can alter activity patterns of neural circuits in the brain and spinal cord. Electrical stimulation may be leveraged following CNS injury to enhance basal excitability of spared circuitry, thereby facilitating supraspinal control or directly activating disconnected networks to initiate activity-associated rewiring or regeneration. Intraspinal microstimulation (ISMS) and dorsal epidural spinal stimulation (ES) are two methods for electrically altering activity in the spinal cord following injury in order to enhance recovery or manage pain. Both ISMS and ES have been applied following cervical injury in rats, and each have proven to be advantageous. Work from the Moritz and Horner laboratories demonstrates beneficial application of ISMS following cervical hemicontusion injury in rats as measured by improvement in a skilled forelimb reaching task following application of cervical stimulation (Sunshine et al., 2013; Mondello et al., 2014). ISMS stimulation of forelimb motor associated motor pools resulted in persistent enhancement of digit extension, pronation, and supination of the wrist during a forelimb reaching task. These effects persisted for hours following cessation of ISMS stimulation (Kasten et al., 2013). Further, cervical ES was beneficial in a cervical crush injury in rats as it reduced aberrant co-activation of antagonistic muscle groups (Alam et al., 2017). After a unilateral pyramidotomy in rats, penetrating electrical stimulation to the forelimb area of the contralateral motor cortex or contralateral medullary pyramid produced increased axon sprouting to the side of the cord ipsilateral to the injury and improved motor function that was still present thirty days after therapeutic electrical stimulation had been halted (Brus-Ramer et al., 2007; Carmel et al., 2010). Electrical stimulation may not only improve plasticity and sprouting, but also affect connectivity and pruning of developing tracts. Stimulation of the medullary pyramid in cats during development maintained connections that might otherwise be pruned and hold the potential to promote sprouting of descending tracts (Salimi and Martin, 2004). New connections are formed spontaneously after midthoracic partial dorsal hemisection, but subsequently lost if they do not connect with intact neurons such as long propriospinal neurons that bridge the lesion site after injury (Bareyre et al., 2004). Thus, electrical stimulation may be critical for maintaining and improving spared spinal circuitry after injury.

In terms of spinal stimulation after SCI, ISMS and ES each have their own advantages; however, they also have drawbacks that limit their therapeutic potential. ISMS allows for relative precision compared to other spinal stimulation paradigms, as intraspinal wires can be guided to specific locations and tested for discrete activity of pools of interest. Given this, ISMS approaches can elicit more controlled modulation of activity in the spinal cord when compared with ES. Importantly, it is not currently feasible to target specific motor pools via dorsal ES. In fact, it is likely that ES preferentially activates central pattern generator circuitry, which limits its therapeutic potential (Bareyre et al., 2004; Taccola et al., 2017) Conversely, scarring and functional stability are major barriers for clinical translation of intraparenchymal stimulation techniques, whereas ES has been widely applied in the clinic with favorable outcomes (Stidd et al., 2014).

Geometric location may be a primary concern when considering ES approaches. Given that sensory and propriospinal afferents are generally located on the dorsal aspect of the cord, while the majority of motor associated circuitry is situated in the ventral region, targeting the ventral surface could allow for more select activation without recruitment of central pattern generators. The ventral surgical approach is commonly applied in humans; however, given the challenge of accessing the ventral epidural space in rodents, ventral epidural spinal stimulation (VSS) has not been extensively studied. Still, an analysis of motor responses in the cervical spine in monkeys revealed that stimulation applied from the ventral and dorsal surfaces evoked muscle responses through different spinal circuitry (Sharpe and Jackson, 2014). Therefore, there is a rationale for exploring geometric placement of epidural electrodes in order to manipulate unique circuits in the spinal cord.

Given the potential for failure and the invasive nature of implantable stimulators, researchers have examined other avenues to therapeutically manipulate neural activity. Techniques such as magnetic stimulation and transcutaneous electrical stimulation are being examined to determine whether less invasive and chronically stable forms of stimulation may facilitate recovery, particularly after SCI, traumatic brain injury, and stroke (Rossini et al., 1994; Hummel and Cohen, 2006; O'Connell et al., 2018).

The idea of non-invasively manipulating neural activity is not a new one. Functional electrical stimulation (FES), a technique of directly stimulating affected musculature, has been leveraged both through implants and via transcutaneous electrodes to facilitate movement and improve function for over five decades. In a meta-analysis of studies applying FES after stroke in humans, FES was found to provide improvement over no intervention and rehabilitative training alone (Howlett et al., 2015). Non-invasive FES is applied through coupled transdermal electrodes placed on the skin that depolarize local motor neurons and facilitate neuromuscular activation and muscle contractions (Peckham and Knutson, 2005). Still, FES is limited in terms of which neurons can be manipulated by stimulation. FES directly activates motor neurons, initiating contractions and ultimately stimulating stretch receptors, thus triggering changes in sensory feedback. This activation is unidirectional, beginning at the motor level and facilitating activity of peripheral nerves, while not directly engaging spinal circuits of motor control. This limitation may represent a ceiling on the potential for FES as a therapy. Still, means of engaging supraspinal and motor associated spinal circuits coincident with FES stimulation exist. Coupling non-invasive EEG recordings with surface stimulating electrodes may provide a means to non-surgically allow “thought-control” of functional movements. This concept has been successfully applied to facilitate task specific movements such as grasping of a cylinder in subjects with tetraplegia (Pfurtscheller et al., 2003). Though closed-loop FES may provide a means to facilitate recruitment of supraspinal and descending motor associated circuits, such techniques are limited in terms of recruiting disconnected circuitry at the spinal and even supraspinal level.

Given this, non-invasive means of applying current directly to the brain and spinal cord have been examined. Transcutaneous electrical stimulation (TES) involves the application of transdermal electrodes above the brain or spinal cord and has been shown to evoke functional activation of motor associated circuitry (Gerasimenko Y. et al., 2015). A major hypothesis behind many TES therapies is that central pattern generator circuits exist in humans and can be accessed when excitability of spinal circuits is raised above a certain level. When a neuromodulatory stimulus is applied, TES can facilitate supraspinal control of muscle activity in humans with motor complete injury and allow strengthening of connections to initiate or improve voluntary control, which would otherwise be impossible (Gerasimenko Y. P. et al., 2015). This motor engagement can lead to functional movements and can even allow for improved postural control (Rath et al., 2018) and voluntary standing without trainer assistance in humans with chronic motor and sensory complete paralysis (Sayenko et al., 2019). Clearly TES can facilitate recruitment of circuits at the spinal level, though a major application barrier is a lack of specificity in terms of circuit engagement. It remains to be shown whether TES can reach deep areas of the brain and spinal cord directly. Regardless, such stimulation will necessarily be non-specific. Further, success of TES is thought to be dependent on spinally encoded circuits and may not extend to more eloquent motor movements outside of stepping and standing. Strategies to raise levels of spinal excitability can lead to spasticity and pain and, as such, more research is needed to understand how TES may be applied safely to improve outcomes after injury.

Transcranial magnetic stimulation (TMS) is another way to non-invasively manipulate activity of neural circuits. It involves generating a magnetic field that creates an electric current to activate neural structures. Since magnetic fields can pass through tissues more readily than electric fields with less interference, they can be used to generate electric currents in deeper brain layers than TES (Walsh and Pascual-Leone, 2003). Resultantly, TMS has been explored in the treatment of depression (Philip et al., 2018), addiction (Diana et al., 2017), migraine headaches (Fischell et al., 2017), traumatic brain injury (Neville et al., 2015), and others. Despite the name, TMS can also be applied to the spine. A study involving twelve human participants with reoccurring neck pain revealed that cervical manipulation of the spine with single and paired TMS pulses resulted in altered corticomotor processing and control of two upper limb muscles, the abductor pollicis brevis and extensor indices proprios (Taylor and Murphy, 2008). Still, the mechanism of action of such therapies is not well-understood. This is in part due to the difficulty of determining exactly which structures and circuits are activated by TMS. *In vitro* studies of cultured rat hippocampal neurons revealed that sensitivity to TMS induction was dependent on the shape and superstructure of neural cultures (Rotem and Moses, 2008). The complicated physics of TMS and our general limited understanding of specifically what may be activated by TMS limits the therapeutic potential of this strategy. Still, the possibility to somewhat selectively activate deeper structures of the brain and spinal cord may be an important component of future therapies.

Indeed, given the importance of selective activation of neural circuitry, novel strategies involving transgenic manipulation of neurons may one day play a therapeutic role. Optogenetics for instance, a technique where a modified algae derived opsin can be genetically engineered into a neuron, allows for wavelength specific activation or inhibition of neurons (Yizhar et al., 2016). Genetic engineering strategies allow local control of specific neural populations through promoter specific transfection and local delivery of an engineered virus. Such strategies have produced techniques that alter neural activity through application of not only light, but also administration of magnetic fields or delivery of generally inert designer drugs (Wheeler et al., 2016; Gomez et al., 2017). While admittedly, such techniques are mostly limited in clinical application, the enhanced targeting of such strategies as well as the ability to selectively initiate changes in specific neural circuits with a high degree of temporal precision will undoubtedly prove a critical resource for uncovering the role of activity-associated plasticity mechanisms at the circuit and cellular level.

## Conclusion

Molecular responses to activity-associated plasticity are driven by a layered and complex system. The mechanisms and outcome of extrinsic or intrinsic changes in activity are context dependent with much to be considered, particularly with regards to the post-injury environment. IEGs drive rapid and transient neuronal responses, which can initiate unique transcription programs following changes in neural activity patterns. The genetic control mechanisms dictating neuronal responses to depolarization patterns result in neural activity pattern-specific genetic expression programs that are circuit and situation specific. While tools to manipulate neural activity have existed for decades, the interplay between neural activity and behavior highlights the importance and potential clinical relevance of new methods to affect both factors synergistically. Given the complexity of interactions, the apparent fluid nature governing cellular responses to changing activity patterns, and the conflicting reported results, more studies are needed to fully realize the clinical applications of activity-associated plasticity. Still, a surge of new tools has led to an impressive growth in our understanding of wide-ranging activity-associated mechanisms and equally driven excitement for the promise of clinical application. Indeed, the partial success of several brain, spine, and peripheral stimulation therapies following trauma or disease hints at the true potential of leveraging activity-associated mechanisms to combat CNS damage. This broad review of activity dependent plasticity, however, reveals the largely siloed nature of laboratories focused on cellular and molecular mechanisms of plasticity and that of research focused on the physiological and functional impact of neuromodulation. Exciting opportunities exist at the intersection of these disciplines that will require tool, model, and conceptual collaborations. Seminal discoveries await that will help us uncover the full extent to which neural activity may be leveraged to provide recovery after CNS injury, degenerative disease, and age-related decline.

## Data Availability Statement

The raw data supporting the conclusions of this article will be made available by the authors, without undue reservation.

## Author Contributions

MH prepared the manuscript with contributions from PH and GH. PH, GH, and MH collected references and materials to present in the review article, and edited the document for correctness and grammar. MH collected and interpreted all data presented in this manuscript, and prepared all drawings and illustrations. All authors contributed to the article and approved the submitted version.

## Conflict of Interest

The authors declare that the research was conducted in the absence of any commercial or financial relationships that could be construed as a potential conflict of interest.

## References

[B1] Adelman K. Lis J. T. (2012). Promoter-proximal pausing of RNA polymerase II: emerging roles in metazoans. Nat. Rev. Genet. 13, 720–731. 10.1038/nrg3293 22986266 PMC3552498

[B2] Ahn S. Ginty D. D. Linden D. J. (1999). A late phase of cerebellar long-term depression requires activation of CaMKIV and CREB. Neuron 23, 559–568. 10.1016/S0896-6273(00)80808-9 10433267

[B3] Alam M. Garcia-Alias G. Jin B. Keyes J. Zhong H. Roy R. R.. (2017). Electrical neuromodulation of the cervical spinal cord facilitates forelimb skilled function recovery in spinal cord injured rats. Exp. Neurol. 291, 141–150. 10.1016/j.expneurol.2017.02.006 28192079 PMC6219872

[B4] Allaman I. Bélanger M. Magistretti P. J. (2011). Astrocyte–neuron metabolic relationships: for better and for worse. Trends Neurosci. 34, 76–87. 10.1016/j.tins.2010.12.001 21236501

[B5] Bading H. (2013). Nuclear calcium signalling in the regulation of brain function. Nat. Rev. Neurosci. 14, 593–608. 10.1038/nrn3531 23942469

[B6] Bahrami S. Drabløs F. (2016). Gene regulation in the immediate-early response process. Adv. Biol. Regul. 62, 37–49. 10.1016/j.jbior.2016.05.001 27220739

[B7] Bareyre F. M. Kerschensteiner M. Raineteau O. Mettenleiter T. C. Weinmann O. Schwab M. E. (2004). The injured spinal cord spontaneously forms a new intraspinal circuit in adult rats. Nat. Neurosci. 7, 269–277. 10.1038/nn1195 14966523

[B8] Berretta A. Tzeng Y.-C. Clarkson A. N. (2014). Post-stroke recovery: the role of activity-dependent release of brain-derived neurotrophic factor. Expert Rev. Neurother. 14, 1335–1344. 10.1586/14737175.2014.969242 25319267

[B9] Brownell J. E. Zhou J. Ranalli T. Kobayashi R. Edmondson D. G. Roth S. Y.. (1996). Tetrahymena histone acetyltransferase A: a homolog to yeast Gcn5p linking histone acetylation to gene activation. Cell 84, 843–851. 10.1016/S0092-8674(00)81063-6 8601308

[B10] Brus-Ramer M. Carmel J. B. Chakrabarty S. Martin J. H. (2007). Electrical stimulation of spared corticospinal axons augments connections with ipsilateral spinal motor circuits after injury. J. Neurosci. 27, 13793–13801. 10.1523/JNEUROSCI.3489-07.2007 18077691 PMC6673617

[B11] Bunch H. Lawney B. P. Lin Y.-F. Asaithamby A. Murshid A. Wang Y. E.. (2015). Transcriptional elongation requires DNA break-induced signalling. Nat. Commun. 6:10191. 10.1038/ncomms10191 26671524 PMC4703865

[B12] Côté M.-P. Azzam G. A. Lemay M. A. Zhukareva V. Houlé J. D. (2011). Activity-dependent increase in neurotrophic factors is associated with an enhanced modulation of spinal reflexes after spinal cord injury. J. Neurotrauma 28, 299–309. 10.1089/neu.2010.1594 21083432 PMC3037803

[B13] Cao L. Jiao X. Zuzga D. S. Liu Y. Fong D. M. Young D.. (2004). VEGF links hippocampal activity with neurogenesis, learning and memory. Nat. Genet. 36, 827–835. 10.1038/ng1395 15258583

[B14] Carmel J. B. Berrol L. J. Brus-Ramer M. Martin J. H. (2010). Chronic electrical stimulation of the intact corticospinal system after unilateral injury restores skilled locomotor control and promotes spinal axon outgrowth. J. Neurosci. 30, 10918–10926. 10.1523/JNEUROSCI.1435-10.2010 20702720 PMC2929360

[B15] Carulli D. Foscarin S. Rossi F. (2011). Activity-dependent plasticity and gene expression modifications in the adult CNS. Front. Mol. Neurosci. 4:50. 10.3389/fnmol.2011.00050 22144945 PMC3226246

[B16] Chan K. M. Curran M. Gordon T. (2016). The use of brief post-surgical low frequency electrical stimulation to enhance nerve regeneration in clinical practice. J. Physiol. 594, 3553–3559. 10.1113/JP270892 26864594 PMC4929315

[B17] Chen J. L. Lin W. C. Cha J. W. So P. T. Kubota Y. Nedivi E. (2011). Structural basis for the role of inhibition in facilitating adult brain plasticity. Nat. Neurosci. 14, 587–594. 10.1038/nn.2799 21478885 PMC3083474

[B18] Chen K. Chen Z. Wu D. Zhang L. Lin X. Su J.. (2015). Broad H3K4me3 is associated with increased transcription elongation and enhancer activity at tumor-suppressor genes. Nat. Genet. 47, 1149–1157. 10.1038/ng.3385 26301496 PMC4780747

[B19] Ciccarelli A. Giustetto M. (2014). Role of ERK signaling in activity-dependent modifications of histone proteins. Neuropharmacology 80, 34–44. 10.1016/j.neuropharm.2014.01.039 24486378

[B20] Cohen T. J. Waddell D. S. Barrientos T. Lu Z. Feng G. Cox G. A.. (2007). The histone deacetylase HDAC4 connects neural activity to muscle transcriptional reprogramming. J. Biol. Chem. 282, 33752–33759. 10.1074/jbc.M706268200 17873280

[B21] Crowe S. L. Movsesyan V. A. Jorgensen T. J. Kondratyev A. (2006). Rapid phosphorylation of histone H2A. X following ionotropic glutamate receptor activation. Eur. J. Neurosci. 23, 2351–2361. 10.1111/j.1460-9568.2006.04768.x 16706843 PMC1534119

[B22] Davis G. W. (2006). Homeostatic control of neural activity: from phenomenology to molecular design. Annu. Rev. Neurosci. 29, 307–323. 10.1146/annurev.neuro.28.061604.135751 16776588

[B23] Dehorter N. Ciceri G. Bartolini G. Lim L. del Pino I. Marín O. (2015). Tuning of fast-spiking interneuron properties by an activity-dependent transcriptional switch. Science 349, 1216–1220. 10.1126/science.aab3415 26359400 PMC4702376

[B24] Diana M. Raij T. Melis M. Nummenmaa A. Leggio L. Bonci A. (2017). Rehabilitating the addicted brain with transcranial magnetic stimulation. Nat. Rev. Neurosci. 18, 685–693. 10.1038/nrn.2017.113 28951609

[B25] Dietz V. Harkema S. J. (2004). Locomotor activity in spinal cord-injured persons. J. Appl. Physiol. 96, 1954–1960. 10.1152/japplphysiol.00942.2003 15075315

[B26] Du J. Feng L. Yang F. Lu B. (2000). Activity-and Ca2+-dependent modulation of surface expression of brain-derived neurotrophic factor receptors in hippocampal neurons. J. Cell Biol. 150, 1423–1434. 10.1083/jcb.150.6.1423 10995446 PMC2150695

[B27] Elzinga K. Tyreman N. Ladak A. Savaryn B. Olson J. Gordon T. (2015). Brief electrical stimulation improves nerve regeneration after delayed repair in sprague dawley rats. Exp. Neurol. 269, 142–153. 10.1016/j.expneurol.2015.03.022 25842267

[B28] Feldman D. E. (2012). The spike-timing dependence of plasticity. Neuron 75, 556–571. 10.1016/j.neuron.2012.08.001 22920249 PMC3431193

[B29] Ferguson A. R. Huie J. R. Crown E. D. Baumbauer K. M. Hook M. A. Garraway S. M. (2012). Maladaptive spinal plasticity opposes spinal learning and recovery in spinal cord injury. Front. Physiol. 3:399 10.3389/fphys.2012.00399 23087647 PMC3468083

[B30] Fields R. D. (2008). Oligodendrocytes changing the rules: action potentials in glia and oligodendrocytes controlling action potentials. Neurosci. 14, 540–543. 10.1177/1073858408320294 19029057 PMC2756778

[B31] Fields R. D. (2015). A new mechanism of nervous system plasticity: activity-dependent myelination. Nat. Rev. Neurosci. 16, 756–767. 10.1038/nrn4023 26585800 PMC6310485

[B32] Fields R. D. Lee P. R. Cohen J. E. (2005). Temporal integration of intracellular Ca2+ signaling networks in regulating gene expression by action potentials. Cell Calcium 37, 433–442. 10.1016/j.ceca.2005.01.011 15820391

[B33] Fields R. D. Nelson P. G. (1992). A role for glial cells in activity-dependent development of the vertebrate nervous system. Int. Rev. Neurobiol. 34, 133–214. 10.1016/S0074-7742(08)60098-7 1587715

[B34] Fischell R. E. Fischell D. R. Fredrick J. P. Woods S. P. (2017). U.S. Patent No. 9,561,384. Washington, DC: U.S. Patent and Trademark Office.

[B35] Flavell S. W. Greenberg M. E. (2008). Signaling mechanisms linking neuronal activity to gene expression and plasticity of the nervous system. Annu. Rev. Neurosci. 31, 563–590. 10.1146/annurev.neuro.31.060407.125631 18558867 PMC2728073

[B36] Fletcher B. R. Calhoun M. E. Rapp P. R. Shapiro M. L. (2006). Fornix lesions decouple the induction of hippocampal arc transcription from behavior but not plasticity. J. Neurosci. 26, 1507–1515. 10.1523/JNEUROSCI.4441-05.2006 16452674 PMC6675482

[B37] Flexner J. B. Flexner L. B. Stellar E. (1963). Memory in mice as affected by intracerebral puromycin. Science 141, 57–59. 10.1126/science.141.3575.57 13945541

[B38] Gariglio P. Bellard M. Chambon P. (1981). Clustering of RNA polymerase B molecules in the 5′ moiety of the adult β-globin gene of hen erythrocytes. Nucleic Acids Res. 9, 2589–2598. 10.1093/nar/9.11.2589 6269056 PMC326874

[B39] Garraway S. M. Huie J. R. (2016). Spinal plasticity and behavior: BDNF-induced neuromodulation in uninjured and injured spinal cord. Neural Plast. 2016:9857201. 10.1155/2016/9857201 27721996 PMC5046018

[B40] Gazula V. Roberts M. Luzzio C. Jawad A. F. Kalb R. G. (2004). Effects of limb exercise after spinal cord injury on motor neuron dendrite structure. J. Comp. Neurol. 476, 130–145. 10.1002/cne.20204 15248194

[B41] Ge S. Pradhan D. A. Ming G. Song H. (2007). GABA sets the tempo for activity-dependent adult neurogenesis. Trends Neurosci. 30, 1–8. 10.1016/j.tins.2006.11.001 17116335

[B42] Gerasimenko Y. Gorodnichev R. Moshonkina T. Sayenko D. Gad P. Edgerton V. R. (2015). Transcutaneous electrical spinal-cord stimulation in humans. Ann. Phys. Rehabil. Med. 58, 225–231. 10.1016/j.rehab.2015.05.003 26205686 PMC5021439

[B43] Gerasimenko Y. P. Lu D. C. Modaber M. Zdunowski S. Gad P. Sayenko D. G.. (2015). Noninvasive reactivation of motor descending control after paralysis. J. Neurotrauma 32, 1968–1980. 10.1089/neu.2015.4008 26077679 PMC4677519

[B44] Ghosh B. Wang Z. Nong J. Urban M. W. Zhang Z. Trovillion V. A.. (2018). Local BDNF delivery to the injured cervical spinal cord using an engineered hydrogel enhances diaphragmatic respiratory function. J. Neurosci. 38, 5982–5995. 10.1523/JNEUROSCI.3084-17.2018 29891731 PMC6021996

[B45] Gomez J. L. Bonaventura J. Lesniak W. Mathews W. B. Sysa-Shah P. Rodriguez L. A.. (2017). Chemogenetics revealed: DREADD occupancy and activation via converted clozapine. Science 357, 503–507. 10.1126/science.aan2475 28774929 PMC7309169

[B46] Gómez-Pinilla F. Ying Z. Roy R. R. Molteni R. Edgerton V. R. (2002). Voluntary exercise induces a BDNF-mediated mechanism that promotes neuroplasticity. J. Neurophysiol. 88, 2187–2195. 10.1152/jn.00152.2002 12424260

[B47] Grau J. W. Huang Y.-J. (2018). Metaplasticity within the spinal cord: Evidence brain-derived neurotrophic factor (BDNF), tumor necrosis factor (TNF), and alterations in GABA function (ionic plasticity) modulate pain and the capacity to learn. Neurobiol. Learn. Mem. 154, 121–135. 10.1016/j.nlm.2018.04.007 29635030 PMC6139037

[B48] Grau J. W. Huang Y.-J. Turtle J. D. Strain M. M. Miranda R. C. Garraway S. M.. (2017). When pain hurts: nociceptive stimulation induces a state of maladaptive plasticity and impairs recovery after spinal cord injury. J. Neurotrauma 34, 1873–1890. 10.1089/neu.2016.4626 27788626 PMC5444485

[B49] Graziano A. Foffani G. Knudsen E. B. Shumsky J. Moxon K. A. (2013). Passive exercise of the hind limbs after complete thoracic transection of the spinal cord promotes cortical reorganization. PLoS ONE 8:e54350. 10.1371/journal.pone.0054350 23349859 PMC3551921

[B50] Guan J.-S. Haggarty S. J. Giacometti E. Dannenberg J.-H. Joseph N. Gao J.. (2009). HDAC2 negatively regulates memory formation and synaptic plasticity. Nature 459, 55–60. 10.1038/nature07925 19424149 PMC3498958

[B51] Guzowski J. F. Miyashita T. Chawla M. K. Sanderson J. Maes L. I. Houston F. P.. (2006). Recent behavioral history modifies coupling between cell activity and arc gene transcription in hippocampal CA1 neurons. Proc. Natl. Acad. Sci. 103, 1077–1082. 10.1073/pnas.0505519103 16415163 PMC1347968

[B52] Guzowski J. F. Timlin J. A. Roysam B. McNaughton B. L. Worley P. F. Barnes C. A. (2005). Mapping behaviorally relevant neural circuits with immediate-early gene expression. Curr. Opin. Neurobiol. 15, 599–606. 10.1016/j.conb.2005.08.018 16150584

[B53] Harkema S. Gerasimenko Y. Hodes J. Burdick J. Angeli C. Chen Y.. (2011). Effect of epidural stimulation of the lumbosacral spinal cord on voluntary movement, standing, and assisted stepping after motor complete paraplegia: a case study. Lancet 377, 1938–1947. 10.1016/S0140-6736(11)60547-3 21601270 PMC3154251

[B54] Hoffman L. R. Field-Fote E. C. (2007). Cortical reorganization following bimanual training and somatosensory stimulation in cervical spinal cord injury: a case report. Phys. Ther. 87, 208–223. 10.2522/ptj.20050365 17213410

[B55] Howlett O. A. Lannin N. A. Ada L. McKinstry C. (2015). Functional electrical stimulation improves activity after stroke: a systematic review with meta-analysis. Arch. Phys. Med. Rehabil. 96, 934–943. 10.1016/j.apmr.2015.01.013 25634620

[B56] Huang Y.-J. Lee K. H. Grau J. W. (2017). Complete spinal cord injury (SCI) transforms how brain derived neurotrophic factor (BDNF) affects nociceptive sensitization. Exp. Neurol. 288, 38–50. 10.1016/j.expneurol.2016.11.001 27818188

[B57] Huang Y.-J. Lee K. H. Murphy L. Garraway S. M. Grau J. W. (2016). Acute spinal cord injury (SCI) transforms how GABA affects nociceptive sensitization. Exp. Neurol. 285, 82–95. 10.1016/j.expneurol.2016.09.005 27639636 PMC5926208

[B58] Hummel F. C. Cohen L. G. (2006). Non-invasive brain stimulation: a new strategy to improve neurorehabilitation after stroke? Lancet Neurol. 5, 708–712. 10.1016/S1474-4422(06)70525-7 16857577

[B59] Im H.-I. Hollander J. A. Bali P. Kenny P. J. (2010). MeCP2 controls BDNF expression and cocaine intake through homeostatic interactions with microRNA-212. Nat. Neurosci. 13, 1120–1127. 10.1038/nn.2615 20711185 PMC2928848

[B60] Inoue Y. Udo H. Inokuchi K. Sugiyama H. (2007). Homer1a regulates the activity-induced remodeling of synaptic structures in cultured hippocampal neurons. Neuroscience 150, 841–852. 10.1016/j.neuroscience.2007.09.081 18006237

[B61] Ji Y. Lu Y. Yang F. Shen W. Tang T. T.-T. Feng L.. (2010). Acute and gradual increases in BDNF concentration elicit distinct signaling and functions in neurons. Nat. Neurosci. 13, 302–309. 10.1038/nn.2505 20173744 PMC4780419

[B62] Jin Y. Fischer I. Tessler A. Houle J. D. (2002). Transplants of fibroblasts genetically modified to express BDNF promote axonal regeneration from supraspinal neurons following chronic spinal cord injury. Exp. Neurol. 177, 265–275. 10.1006/exnr.2002.7980 12429228

[B63] Jolivet R. Coggan J. S. Allaman I. Magistretti P. J. (2015). Multi-timescale modeling of activity-dependent metabolic coupling in the neuron-glia-vasculature ensemble. PLoS Comput. Biol. 11:e1004036. 10.1371/journal.pcbi.1004036 25719367 PMC4342167

[B64] Jonkers I. Lis J. T. (2015). Getting up to speed with transcription elongation by RNA polymerase II. Nat. Rev. Mol. Cell Biol. 16, 167–177. 10.1038/nrm3953 25693130 PMC4782187

[B65] Joo J.-Y. Schaukowitch K. Farbiak L. Kilaru G. Kim T.-K. (2016). Stimulus-specific combinatorial functionality of neuronal c-fos enhancers. Nat. Neurosci. 19, 75–83. 10.1038/nn.4170 26595656 PMC4696896

[B66] Jurkiewicz M. T. Mikulis D. J. McIlroy W. E. Fehlings M. G. Verrier M. C. (2007). Sensorimotor cortical plasticity during recovery following spinal cord injury: a longitudinal fMRI study. Neurorehabil. Neural Repair 21, 527–538. 10.1177/1545968307301872 17507643

[B67] Kandel E. R. (2001). The molecular biology of memory storage: a dialogue between genes and synapses. Science 294, 1030–1038. 10.1126/science.1067020 11691980

[B68] Kao T. Shumsky J. S. Knudsen E. B. Murray M. Moxon K. A. (2011). Functional role of exercise-induced cortical organization of sensorimotor cortex after spinal transection. J. Neurophysiol. 106, 2662–2674. 10.1152/jn.01017.2010 21865438 PMC3214119

[B69] Karpova N. N. (2014). Role of BDNF epigenetics in activity-dependent neuronal plasticity. Neuropharmacology 76, 709–718. 10.1016/j.neuropharm.2013.04.002 23587647

[B70] Kasten M. R. Sunshine M. D. Secrist E. S. Horner P. J. Moritz C. T. (2013). Therapeutic intraspinal microstimulation improves forelimb function after cervical contusion injury. J. Neural Eng. 10:44001. 10.1088/1741-2560/10/4/044001 23715242 PMC3748939

[B71] Kellner Y. Gödecke N. Dierkes T. Thieme N. Zagrebelsky M. Korte M. K. (2014). The BDNF effects on dendritic spines of mature hippocampal neurons depend on neuronal activity. Front. Synaptic Neurosci. 6:5. 10.3389/fnsyn.2014.00005 24688467 PMC3960490

[B72] Kim T.-K. Hemberg M. Gray J. M. Costa A. M. Bear D. M. Wu J.. (2010). Widespread transcription at neuronal activity-regulated enhancers. Nature 465, 182–187. 10.1038/nature09033 20393465 PMC3020079

[B73] Kiser T. S. Reese N. B. Maresh T. Hearn S. Yates C. Skinner R.. (2005). Use of a motorized bicycle exercise trainer to normalize frequency-dependent habituation of the H-reflex in spinal cord injury. J. Spinal Cord Med. 28, 241–245. 10.1080/10790268.2005.11753818 16048142

[B74] Kondiles B. R. Horner P. J. (2018). Myelin plasticity, neural activity, and traumatic neural injury. Dev. Neurobiol. 78, 108–122. 10.1002/dneu.22540 28925069

[B75] Koppel I. Timmusk T. (2013). Differential regulation of Bdnf expression in cortical neurons by class-selective histone deacetylase inhibitors. Neuropharmacology 75, 106–115. 10.1016/j.neuropharm.2013.07.015 23916482

[B76] Kravitz A. Bonci A. (2013). Optogenetics, physiology, and emotions. Front. Behav. Neurosci. 7:169. 10.3389/fnbeh.2013.00169 24312032 PMC3833017

[B77] Lai K.-O. Wong A. S. L. Cheung M.-C. Xu P. Liang Z. Lok K.-C.. (2012). TrkB phosphorylation by Cdk5 is required for activity-dependent structural plasticity and spatial memory. Nat. Neurosci. 15, 1506–1515. 10.1038/nn.3237 23064382 PMC7511999

[B78] Lavrov I. Dy C. J. Fong A. J. Gerasimenko Y. Courtine G. Zhong H.. (2008). Epidural stimulation induced modulation of spinal locomotor networks in adult spinal rats. J. Neurosci. 28, 6022–6029. 10.1523/JNEUROSCI.0080-08.2008 18524907 PMC2904311

[B79] Lee P. R. Cohen J. E. Iacobas D. A. Iacobas S. Fields R. D. (2017). Gene networks activated by specific patterns of action potentials in dorsal root ganglia neurons. Sci. Rep. 7:43765. 10.1038/srep43765 28256583 PMC5335607

[B80] Leech K. A. Hornby T. G. (2017). High-intensity locomotor exercise increases brain-derived neurotrophic factor in individuals with incomplete spinal cord injury. J. Neurotrauma 34, 1240–1248. 10.1089/neu.2016.4532 27526567 PMC5359683

[B81] Li X. Marshall P. R. Leighton L. J. Zajaczkowski E. L. Wang Z. Madugalle S. U.. (2019). The DNA repair-associated protein Gadd45γ regulates the temporal coding of immediate early gene expression within the prelimbic prefrontal cortex and is required for the consolidation of associative fear memory. J. Neurosci. 39, 970–983. 10.1523/JNEUROSCI.2024-18.2018 30545945 PMC6363930

[B82] Linderoth B. Foreman R. D. (1999). Physiology of spinal cord stimulation: review and update. Neuromudulation 2, 150–164. 10.1046/j.1525-1403.1999.00150.x 22151202

[B83] Liu J. Wu X. Zhang H. Pfeifer G. P. Lu Q. (2017). Dynamics of RNA polymerase II pausing and bivalent histone H3 methylation during neuronal differentiation in brain development. Cell Rep. 20, 1307–1318. 10.1016/j.celrep.2017.07.046 28793256 PMC5564459

[B84] Liu K. Tedeschi A. Park K. K. He Z. (2011). Neuronal intrinsic mechanisms of axon regeneration. Annu. Rev. Neurosci. 34, 131–152. 10.1146/annurev-neuro-061010-113723 21438684

[B85] Lu Y. Christian K. Lu B. (2008). BDNF: a key regulator for protein synthesis-dependent LTP and long-term memory? Neurobiol. Learn. Mem. 89, 312–323. 10.1016/j.nlm.2007.08.018 17942328 PMC2387254

[B86] Lynskey J. V. Belanger A. Jung R. (2008). Activity-dependent plasticity in spinal cord injury. J. Rehabil. Res. Dev. 45, 229–40. 10.1682/JRRD.2007.03.0047 18566941 PMC2562625

[B87] Ma D. K. Jang M.-H. Guo J. U. Kitabatake Y. Chang M. Pow-Anpongkul N.. (2009). Neuronal activity–induced Gadd45b promotes epigenetic DNA demethylation and adult neurogenesis. Science 323, 1074–1077. 10.1126/science.1166859 19119186 PMC2726986

[B88] Madabhushi R. Gao F. Pfenning A. R. Pan L. Yamakawa S. Seo J.. (2015). Activity-induced DNA breaks govern the expression of neuronal early-response genes. Cell 161, 1592–1605. 10.1016/j.cell.2015.05.032 26052046 PMC4886855

[B89] Madabhushi R. Kim T.-K. (2018). Emerging themes in neuronal activity-dependent gene expression. Mol. Cell. Neurosci. 87, 27–34. 10.1016/j.mcn.2017.11.009 29254824 PMC5894330

[B90] Mailis-Gagnon A. Furlan A. D. Sandoval J. A. Taylor R. S. (2004). Spinal cord stimulation for chronic pain. Cochrane Database Syst Rev. Rev. 3, 1454–1858. 10.1002/14651858.CD003783.pub2 15266501

[B91] Marais L. Stein D. J. Daniels W. M. U. (2009). Exercise increases BDNF levels in the striatum and decreases depressive-like behavior in chronically stressed rats. Metab. Brain Dis. 24, 587–597. 10.1007/s11011-009-9157-2 19844781

[B92] Martin J. H. (2005). The corticospinal system: from development to motor control. Neurosci. 11, 161–173. 10.1177/1073858404270843 15746384

[B93] Medina I. Friedel P. Rivera C. Kahle K. T. Kourdougli N. Uvarov P.. (2014). Current view on the functional regulation of the neuronal K+-Cl– cotransporter KCC2. Front. Cell. Neurosci. 8:27. 10.3389/fncel.2014.00027 24567703 PMC3915100

[B94] Minatohara K. Akiyoshi M. Okuno H. (2016). Role of immediate-early genes in synaptic plasticity and neuronal ensembles underlying the memory trace. Front. Mol. Neurosci. 8:78. 10.3389/fnmol.2015.00078 26778955 PMC4700275

[B95] Mondello S. E. Kasten M. R. Horner P. J. Moritz C. T. (2014). Therapeutic intraspinal stimulation to generate activity and promote long-term recovery. Front. Neurosci. 8:21. 10.3389/fnins.2014.00021 24578680 PMC3936503

[B96] Morgan J. I. Curran T. (1986). Role of ion flux in the control of c-fos expression. Nature 322, 552–555. 10.1038/322552a0 2426600

[B97] Morgan J. I. Curran T. (1989). Stimulus-transcription coupling in neurons: role of cellular immediate-early genes. Trends Neurosci. 12, 459–462. 10.1016/0166-2236(89)90096-9 2479148

[B98] Naeve G. S. Ramakrishnan M. Kramer R. Hevroni D. Citri Y. Theill L. E. (1997). Neuritin: a gene induced by neural activity and neurotrophins that promotes neuritogenesis. Proc. Natl. Acad. Sci. U.S.A. 94, 2648–2653. 10.1073/pnas.94.6.2648 9122250 PMC20143

[B99] Nagahara A. H. Tuszynski M. H. (2011). Potential therapeutic uses of BDNF in neurological and psychiatric disorders. Nat. Rev. Drug Discov. 10, 209–219. 10.1038/nrd3366 21358740

[B100] Nagappan G. Lu B. (2005). Activity-dependent modulation of the BDNF receptor TrkB: mechanisms and implications. Trends Neurosci. 28, 464–471. 10.1016/j.tins.2005.07.003 16040136

[B101] Neville I. S. Hayashi C. Y. El Hajj S. A. Zaninotto A. L. C. Sabino J. P. Sousa L. M.. (2015). Repetitive transcranial magnetic stimulation (rTMS) for the cognitive rehabilitation of traumatic brain injury (TBI) victims: study protocol for a randomized controlled trial. Trials 16:440. 10.1186/s13063-015-0944-2 26438108 PMC4594992

[B102] O'Connell N. E. Marston L. Spencer S. DeSouza L. H. Wand B. M. (2018). Non-invasive brain stimulation techniques for chronic pain. Cochrane Database Syst Rev. 8:CD008208 10.1002/14651858.CD008208.pub5 PMC6494527 29652088

[B103] Okuno H. (2011). Regulation and function of immediate-early genes in the brain: beyond neuronal activity markers. Neurosci. Res. 69, 175–186. 10.1016/j.neures.2010.12.007 21163309

[B104] Palomer E. Carretero J. Benvegnu S. Dotti C. G. Martin M. G. (2016). Neuronal activity controls Bdnf expression via polycomb de-repression and CREB/CBP/JMJD3 activation in mature neurons. Nat. Commun. 7:11081. 10.1038/ncomms11081 27010597 PMC4820842

[B105] Pastuzyn E. D. Day C. E. Kearns R. B. Kyrke-Smith M. Taibi A. V. McCormick J.. (2018). The neuronal gene Arc encodes a repurposed retrotransposon Gag protein that mediates intercellular RNA transfer. Cell 172, 275–288. 10.1016/j.cell.2017.12.024 29328916 PMC5884693

[B106] Peckham P. H. Knutson J. S. (2005). Functional electrical stimulation for neuromuscular applications. Annu. Rev. Biomed. Eng. 7, 327–360. 10.1146/annurev.bioeng.6.040803.140103 16004574

[B107] Pfurtscheller G. Müller G. R. Pfurtscheller J. Gerner H. J. Rupp R. (2003). ‘Thought'–control of functional electrical stimulation to restore hand grasp in a patient with tetraplegia. Neurosci. Lett. 351, 33–36. 10.1016/S0304-3940(03)00947-9 14550907

[B108] Philip N. S. Barredo J. Aiken E. Carpenter L. L. (2018). Neuroimaging mechanisms of therapeutic transcranial magnetic stimulation for major depressive disorder. Biol. Psychiatry Cogn. Neurosci. Neuroimaging 3, 211–222. 10.1016/j.bpsc.2017.10.007 29486862 PMC5856477

[B109] Qiu Z. Ghosh A. (2008). A calcium-dependent switch in a CREST-BRG1 complex regulates activity-dependent gene expression. Neuron 60, 775–787. 10.1016/j.neuron.2008.09.040 19081374 PMC2615455

[B110] Rasmussen P. Brassard P. Adser H. Pedersen M. V. Leick L. Hart E.. (2009). Evidence for a release of brain-derived neurotrophic factor from the brain during exercise. Exp. Physiol. 94, 1062–1069. 10.1113/expphysiol.2009.048512 19666694

[B111] Rath M. Vette A. H. Ramasubramaniam S. Li K. Burdick J. Edgerton V. R.. (2018). Trunk stability enabled by noninvasive spinal electrical stimulation after spinal cord injury. J. Neurotrauma 35, 2540–2553. 10.1089/neu.2017.5584 29786465 PMC6205803

[B112] Rea S. Eisenhaber F. O'carroll D. Strahl B. D. Sun Z.-W. Schmid M.. (2000). Regulation of chromatin structure by site-specific histone H3 methyltransferases. Nature 406, 593–599. 10.1038/35020506 10949293

[B113] Rebesco J. M. Stevenson I. H. Koerding K. Solla S. A. Miller L. E. (2010). Rewiring neural interactions by micro-stimulation. Front. Syst. Neurosci. 4:39. 10.3389/fnsys.2010.00039 20838477 PMC2936935

[B114] Rendeiro C. Rhodes J. S. (2018). A new perspective of the hippocampus in the origin of exercise–brain interactions. Brain Struct. Funct. 223, 2527–2545. 10.1007/s00429-018-1665-6 29671055

[B115] Rösche J. Paulus C. Maisch U. Kaspar A. Mauch E. Kornhuber H. H. (1997). The effects of therapy on spasticity utilizing a motorized exercise-cycle. Spinal Cord 35, 176–178. 10.1038/sj.sc.3100376 9076869

[B116] Rossini P. M. Barker A. T. Berardelli A. Caramia M. D. Caruso G. Cracco R. Q.. (1994). Non-invasive electrical and magnetic stimulation of the brain, spinal cord and roots: basic principles and procedures for routine clinical application. Report of an IFCN committee. Electroencephalogr. Clin. Neurophysiol. 91, 79–92. 10.1016/0013-4694(94)90029-9 7519144

[B117] Rotem A. Moses E. (2008). Magnetic stimulation of one-dimensional neuronal cultures. Biophys. J. 94, 5065–5078. 10.1529/biophysj.107.125708 18326634 PMC2397342

[B118] Sakry D. Neitz A. Singh J. Frischknecht R. Marongiu D. Biname F.. (2014). Oligodendrocyte precursor cells modulate the neuronal network by activity-dependent ectodomain cleavage of glial NG2. PLoS Biol. 12:e1001993. 10.1371/journal.pbio.1001993 25387269 PMC4227637

[B119] Salimi I. Martin J. H. (2004). Rescuing transient corticospinal terminations and promoting growth with corticospinal stimulation in kittens. J. Neurosci. 24, 4952–4961. 10.1523/JNEUROSCI.0004-04.2004 15163687 PMC6729378

[B120] Sayenko D. G. Rath M. Ferguson A. R. Burdick J. W. Havton L. A. Edgerton V. R.. (2019). Self-assisted standing enabled by non-invasive spinal stimulation after spinal cord injury. J. Neurotrauma 36, 1435–1450. 10.1089/neu.2018.5956 30362876 PMC6482915

[B121] Schafer D. P. Lehrman E. K. Kautzman A. G. Koyama R. Mardinly A. R. Yamasaki R.. (2012). Microglia sculpt postnatal neural circuits in an activity and complement-dependent manner. Neuron 74, 691–705. 10.1016/j.neuron.2012.03.026 22632727 PMC3528177

[B122] Schaukowitch K. Joo J.-Y. Liu X. Watts J. K. Martinez C. Kim T.-K. (2014). Enhancer RNA facilitates NELF release from immediate early genes. Mol. Cell 56, 29–42. 10.1016/j.molcel.2014.08.023 25263592 PMC4186258

[B123] Seeman S. C. Mogen B. J. Fetz E. E. Perlmutter S. I. (2017). Paired stimulation for spike-timing-dependent plasticity in primate sensorimotor cortex. J. Neurosci. 37, 1935–1949. 10.1523/JNEUROSCI.2046-16.2017 28093479 PMC5320619

[B124] Sharpe A. N. Jackson A. (2014). Upper-limb muscle responses to epidural, subdural and intraspinal stimulation of the cervical spinal cord. J. Neural Eng. 11:16005. 10.1088/1741-2560/11/1/016005 24654267 PMC4013994

[B125] Sheng M. Dougan S. T. McFadden G. Greenberg M. E. (1988). Calcium and growth factor pathways of c-fos transcriptional activation require distinct upstream regulatory sequences. Mol. Cell. Biol. 8, 2787–2796. 10.1128/MCB.8.7.2787 3136322 PMC363496

[B126] Sheng M. Greenberg M. E. (1990). The regulation and function of c-fos and other immediate early genes in the nervous system. Neuron 4, 477–485. 10.1016/0896-6273(90)90106-P 1969743

[B127] Sheng M. Thompson M. A. Greenberg M. E. (1991). CREB: a Ca (2+)-regulated transcription factor phosphorylated by calmodulin-dependent kinases. Science 252, 1427–1430. 10.1126/science.1646483 1646483

[B128] Shimada T. Yoshida T. Yamagata K. (2016). Neuritin mediates activity-dependent axonal branch formation in part via FGF signaling. J. Neurosci. 36, 4534–4548. 10.1523/JNEUROSCI.1715-15.2016 27098696 PMC6601825

[B129] Skinner R. D. Houle J. D. Reese N. B. Berry C. L. Garcia-Rill E. (1996). Effects of exercise and fetal spinal cord implants on the H-reflex in chronically spinalized adult rats. Brain Res. 729, 127–131. 10.1016/0006-8993(96)00556-2 8874885

[B130] Sleiman S. F. Henry J. Al-Haddad R. El Hayek L. Haidar E. A. Stringer T.. (2016). Exercise promotes the expression of brain derived neurotrophic factor (BDNF) through the action of the ketone body β-hydroxybutyrate. Elife 5:e15092. 10.7554/eLife.15092.012 27253067 PMC4915811

[B131] Smith P. A. (2014). BDNF: no gain without pain? Neuroscience 283, 107–123. 10.1016/j.neuroscience.2014.05.044 24887639

[B132] Spiegel I. Mardinly A. R. Gabel H. W. Bazinet J. E. Couch C. H. Tzeng C. P.. (2014). Npas4 regulates excitatory-inhibitory balance within neural circuits through cell-type-specific gene programs. Cell 157, 1216–1229. 10.1016/j.cell.2014.03.058 24855953 PMC4089405

[B133] Spitzer N. C. (2006). Electrical activity in early neuronal development. Nature 444, 707–712. 10.1038/nature05300 17151658

[B134] Stagg C. J. Nitsche M. A. (2011). Physiological basis of transcranial direct current stimulation. Neuroscientist 17, 37–53. 10.1177/1073858410386614 21343407

[B135] Stidd D. A. Rivero S. Weinand M. E. (2014). Spinal cord stimulation with implanted epidural paddle lead relieves chronic axial low back pain. J. Pain Res. 7, 465–470. 10.2147/JPR.S66414 25143753 PMC4136982

[B136] Stimpson N. J. Davison G. Javadi A.-H. (2018). Joggin'the noggin: towards a physiological understanding of exercise-induced cognitive benefits. Neurosci. Biobehav. Rev. 88, 177–186. 10.1016/j.neubiorev.2018.03.018 29572187

[B137] Suberbielle E. Sanchez P. E. Kravitz A. V. Wang X. Ho K. Eilertson K.. (2013). Physiologic brain activity causes DNA double-strand breaks in neurons, with exacerbation by amyloid-β. Nat. Neurosci. 16, 613–621. 10.1038/nn.3356 23525040 PMC3637871

[B138] Sunshine M. D. Cho F. S. Lockwood D. R. Fechko A. S. Kasten M. R. Moritz C. T. (2013). Cervical intraspinal microstimulation evokes robust forelimb movements before and after injury. J. Neural Eng. 10:36001. 10.1088/1741-2560/10/3/036001 23548462 PMC3732065

[B139] Taccola G. (2011). The locomotor central pattern generator of the rat spinal cord in vitro is optimally activated by noisy dorsal root waveforms. J. Neurophysiol. 106, 872–884. 10.1152/jn.00170.2011 21613591

[B140] Taccola G. Sayenko D. Gad P. Gerasimenko Y. Edgerton V. R. (2017). And yet it moves: Recovery of volitional control after spinal cord injury. Prog Neurobiol. 160:64–81. 10.1016/j.pneurobio.2017.10.004 29102670 PMC5773077

[B141] Taylor H. H. Murphy B. (2008). Altered sensorimotor integration with cervical spine manipulation. J. Manipulative Physiol. Ther. 31, 115–126. 10.1016/j.jmpt.2007.12.011 18328937

[B142] Tongiorgi E. Righi M. Cattaneo A. (1997). Activity-dependent dendritic targeting of BDNF and TrkB mRNAs in hippocampal neurons. J. Neurosci. 17, 9492–9505. 10.1523/JNEUROSCI.17-24-09492.1997 9391005 PMC6573421

[B143] Tyssowski K. M. DeStefino N. R. Cho J.-H. Dunn C. J. Poston R. G. Carty C. E.. (2018). Different neuronal activity patterns induce different gene expression programs. Neuron 98, 530–546. 10.1016/j.neuron.2018.04.001 29681534 PMC5934296

[B144] Walsh V. Pascual-Leone A. (2003). Transcranial Magnetic Stimulation: A Neurochronometrics of Mind. Cambridge, MA: MIT press 10.7551/mitpress/6896.001.0001

[B145] Wheeler M. A. Smith C. J. Ottolini M. Barker B. S. Purohit A. M. Grippo R. M.. (2016). Genetically targeted magnetic control of the nervous system. Nat. Neurosci. 19, 756–761. 10.1038/nn.4265 26950006 PMC4846560

[B146] Williams A. Gill S. Varma T. Jenkinson C. Quinn N. Mitchell R.. (2010). Deep brain stimulation plus best medical therapy versus best medical therapy alone for advanced parkinson's disease (PD SURG trial): a randomised, open-label trial. Lancet Neurol. 9, 581–591. 10.1016/S1474-4422(10)70093-4 20434403 PMC2874872

[B147] Wong Y.-H. Lee C.-M. Xie W. Cui B. Poo M. (2015). Activity-dependent BDNF release via endocytic pathways is regulated by synaptotagmin-6 and complexin. Proc. Natl. Acad. Sci. U.S.A. 112, E4475–E4484. 10.1073/pnas.1511830112 26216953 PMC4538679

[B148] Xiao B. Tu J. C. Worley P. F. (2000). Homer: a link between neural activity and glutamate receptor function. Curr. Opin. Neurobiol. 10, 370–374. 10.1016/S0959-4388(00)00087-8 10851183

[B149] Xiao J. Wong A. W. Willingham M. M. Kaasinen S. K. Hendry I. A. Howitt J.. (2009). BDNF exerts contrasting effects on peripheral myelination of NGF-dependent and BDNF-dependent DRG neurons. J. Neurosci. 29, 4016–4022. 10.1523/JNEUROSCI.3811-08.2009 19339597 PMC6665359

[B150] Yao J. Zhao Q. Lu J. Mei Y. (2018). Functions and the related signaling pathways of the neurotrophic factor neuritin. Acta Pharmacol. Sin. 39, 1414–1420. 10.1038/aps.2017.197 29595190 PMC6289377

[B151] Yao Y.-X. Zhang Y.-F. Yang Y. Guo S.-H. Jiang Z. Zhao Z.-Q. (2014). Spinal synaptic scaffolding protein Homer 1b/c regulates CREB phosphorylation and c-fos activation induced by inflammatory pain in rats. Neurosci. Lett. 559, 88–93. 10.1016/j.neulet.2013.11.049 24316406

[B152] Yizhar O. Fenno L. E. Davidson T. J. Mogri M. Deisseroth K. (2016). Optogenetics in Neural Systems. Neuron 71, 9–34. 10.1016/j.neuron.2011.06.004 21745635

[B153] Young S. Z. Taylor M. M. Bordey A. (2011). Neurotransmitters couple brain activity to subventricular zone neurogenesis. Eur. J. Neurosci. 33, 1123–1132. 10.1111/j.1460-9568.2011.07611.x 21395856 PMC3075963

[B154] Zagrebelsky M. Korte M. (2014). Form follows function: BDNF and its involvement in sculpting the function and structure of synapses. Neuropharmacology 76, 628–638. 10.1016/j.neuropharm.2013.05.029 23752094

[B155] Zanos S. Richardson A. G. Shupe L. Miles F. P. Fetz E. E. (2011). The Neurochip-2: an autonomous head-fixed computer for recording and stimulating in freely behaving monkeys. IEEE Trans. Neural Syst. Rehabil. Eng. 19, 427–435. 10.1109/TNSRE.2011.2158007 21632309 PMC3159515

[B156] Zhao Q. Lu J. Li Z. Mei Y. (2018). Neuritin promotes neurite and spine growth in rat cerebellar granule cells via L-type calcium channel-mediated calcium influx. J. Neurochem. 147, 40–57. 10.1111/jnc.14535 29920676 PMC6220818

[B157] Zhou Z. Hong E. J. Cohen S. Zhao W. Ho H. H. Schmidt L.. (2006). Brain-specific phosphorylation of MeCP2 regulates activity-dependent Bdnf transcription, dendritic growth, and spine maturation. Neuron 52, 255–269. 10.1016/j.neuron.2006.09.037 17046689 PMC3962021

